# Selective sorption of oxygen and nitrous oxide by an electron donor-incorporated flexible coordination network

**DOI:** 10.1038/s42004-023-00853-1

**Published:** 2023-04-04

**Authors:** Mohana Shivanna, Jia-Jia Zheng, Keith G. Ray, Sho lto, Hirotaka Ashitani, Yoshiki Kubota, Shogo Kawaguchi, Vitalie Stavila, Ming-Shui Yao, Takao Fujikawa, Ken-ichi Otake, Susumu Kitagawa

**Affiliations:** 1 Institute for Integrated Cell-Material Sciences, Kyoto University Institute for Advanced Study, Kyoto University, Yoshida Ushinomiya-cho, Sakyo-ku, Kyoto, 606-8501 Japan; 2 Laboratory of Theoretical and Computational Nanoscience, CAS Center for Excellence in Nanoscience, National Center for Nanoscience and Technology, Chinese Academy of Sciences, No. 11 ZhongGuanCun BeiYiTiao, Beijing, 100190 P. R. China; 3 Lawrence Livermore National Laboratory, Livermore, CA 94550 USA; 4 Rigaku Corporation, 3-9-12 Matsubara-cho, Akishima, Tokyo 196-8666 Japan; 5 Department of Physics, Graduate School of Science, Osaka Prefecture University, Sakai, Osaka, 599-8531 Japan; 6 Department of Physical Science, Graduate School of Science, Osaka Metropolitan University, Sakai, Osaka, 599-8531 Japan; 7 Japan Synchrotron Radiation Research Institute (JASRI), SPring-8, 1-1-1 Kouto, Sayo-cho, Sayo-gun, Hyogo, 679-5198 Japan; 8 Sandia National Laboratory, Livermore, CA 94550 USA

**Keywords:** Porous materials, Metal-organic frameworks, Coordination chemistry

## Abstract

Incorporating strong electron donor functionality into flexible coordination networks is intriguing for sorption applications due to a built-in mechanism for electron-withdrawing guests. Here we report a 2D flexible porous coordination network, [Ni_2_(4,4′-bipyridine)(VTTF)_2_]n(1) (where H_2_VTTF = 2,2′-[1,2-bis(4-benzoic acid)-1,2ethanediylidene]bis-1,3-benzodithiole), which exhibits large structural deformation from the as-synthesized or open phase (1α) into the closed phase (1β) after guest removal, as demonstrated by X-ray and electron diffraction. Interestingly, upon exposure to electron-withdrawing species, 1β reversibly undergoes guest accommodation transitions; 1α⊃O_2_ (90 K) and 1α⊃N_2_O (185 K). Moreover, the 1β phase showed exclusive O_2_ sorption over other gases (N_2_, Ar, and CO) at 120 K. The phase transformations between the 1α and 1β phases under these gases were carefully investigated by in-situ X-ray diffraction, in-situ spectroscopic studies, and DFT calculations, validating that the unusual sorption was attributed to the combination of flexible frameworks and VTTF (electron-donor) that induces strong interactions with electron-withdrawing species.

## Abstract (web summary)

Incorporating electron donor functionalities into porous coordination frameworks enables the strong binding of electron-withdrawing guests, but such binding typically occurs irreversibly. Here, a structurally dynamic 2D coordination network incorporating an electron donating group is found to selectively and reversibly bind oxygen and nitrous oxide, while also exhibiting large structural deformations after guest removal.

## Introduction

Porous coordination polymers (PCPs)^1,2^ or metal-organic frameworks (MOFs)^3,4^ are an emerging class of solid-state materials composed of metal or metal cluster-based nodes linked by various multifunctional organic linkers. They are extraordinarily diverse in composition because of their modularity and amenability to rational design principles^5^. A small subset of PCPs exhibits structural transformations when exposed to external stimuli, such as heat, light, and pressure^6,7^. Such flexible or soft PCP provides a superior adsorption system, not available with conventional materials, with highly selective recognition properties toward weakly-interacting gas molecules, for example, CO_2_/CH_4_^8^, C2^6,9^, C3^10,11^, and other higher hydrocarbons^12,13^. In addition to their size and shape-conforming effects^13,14^, these soft PCPs are proving to be helpful for a variety of adsorption applications^15–17^, taking advantage of the electron-donating and attraction-based interactions of the guest molecules^18,19^. The development of selective adsorbents, especially for the valuable and versatile O_2_, requires the creation of PCPs that integrate the following strategies; (i) the incorporation of Lewis acidic sites into a robust framework^20–25^, (ii) the use of the optimized pore size or shape to accommodate O_2_ molecules^26^, and (iii) the use of flexible frameworks^27–29^. Among these, strategy (i) is the most commonly used because some PCPs have strong O_2_ binding sites originating from coordinatively unsaturated metal ions (open metal sites). However, they often suffer from the irreversibility of the O_2_ binding. For example, Fe_2_(dobdc)^20^ preferentially binds O_2_ over N_2_, with a reversible capacity of 18.2 wt% at 211 K. However, above 298 K, it showed irreversible adsorption behavior because of the covalent binding of O_2_ at the open metal sites. (ii) The use of optimized pore size or shape for O_2_ molecules, which often suffer from the co-adsorption with similar dimension-possessing gases, has also been reported^20–26^. The combination of the redox property and a flexible framework of [Zn(TCNQ–TCNQ)bpy] (TCNQ = 7,7,8,8‐tetracyanquinonedimethane)^30,31^ gives hints for the development of adsorbents with higher oxygen selectivity. However, this PCP required cryogenic temperature (77 K) for the selective O_2_ sorption, which is lower than oxygen boiling point (90 K). Moreover, due to the relatively unstable TCNQ dimer unit in the framework, this PCP decomposes at around 200 °C^30^.

**Fig. 1:**
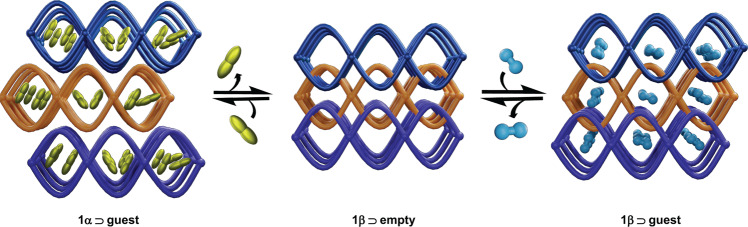
Schematic representation of structural transformations upon gas adsorption. The 1β⊃empty phase reversibly transforms to 1α⊃O_2_ at 90 K, 1α⊃N_2_O at 185 K, and 1α⊃C_6_H_6_ at 298 K (left). When O_2_ sorption was measured at slightly higher temperatures (120 K), 1β⊃empty adsorb selectively O_2_ and undergoes to 1β⊃O_2_ with slight distortion in the 2D network (right).

In this study, we synthesized a stable flexible porous coordination network, [Ni_2_(VTTF)_2_(4,4’-bipyridine)]_*n*_ (H_2_VTTF = 2,2′-[1,2-bis(4-benzoic acid)-1,2ethanediylidene]bis-1,3-benzodithiole), which has a 2D network structure constructed from the donor-type VTTF ligands. This framework exhibits gate-opening behavior selectively for O_2_ and N_2_O over N_2_, Ar, H_2_, CH_4_, and CO_2_ because of its flexibility and electron-rich donor properties (Fig. 1 and Supplementary Table 1). The mechanism of the selective recognition behavior was carefully investigated using electron diffraction, in-situ X-ray diffraction, in-situ spectroscopic studies, and density functional theory calculations, confirming that superb sorption was due to the combination of flexible frameworks and vinylogous tetrathiafulvalene (electron donor) that induces strong host–guest interactions with electron-withdrawing species.

## Results and discussions

### Synthesis and crystal structures

**Fig. 2:**
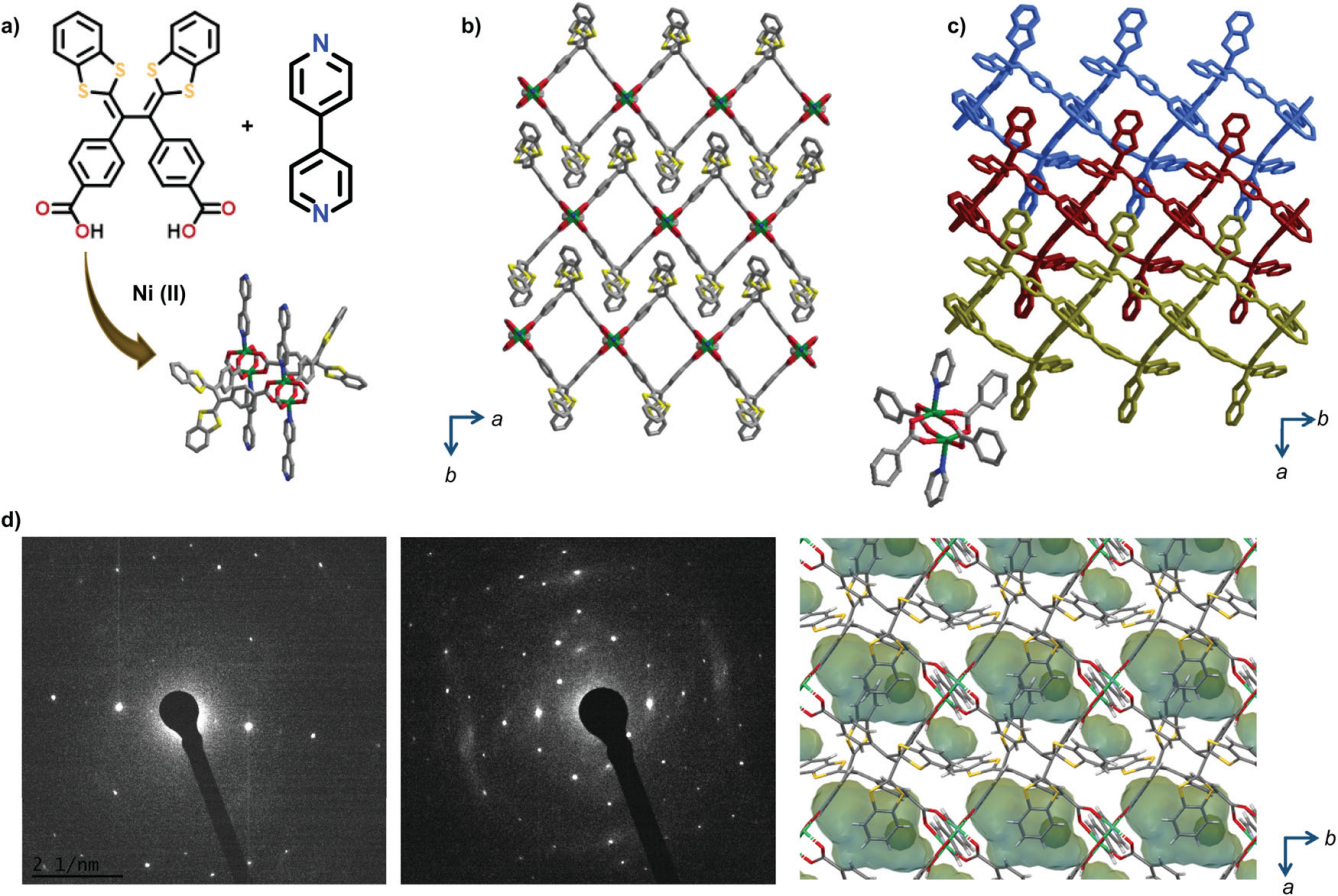
Structural representation. **a** Chemical structure of organic and inorganic components to construct a 2D layered net. **b** closed packed structure of 1α phase, guest accessible channels (49%), and **c** 1β⊃empty phase structure obtained after thermal treatment or guest exchange to form discrete voids (13%). **d** The selected area electron diffraction (SAED) images obtained for 1β from the local regions on TEM.

A 2D layered coordination network, [Ni_2_(VTTF)_2_(4,4’-bipyridine)]_n_, was prepared by the solvothermal reaction between Ni(NO_3_)_2_·6H_2_O, 4,4’-bipyridine and H_2_VTTF, using N, N’-dimethylformamide (DMF), and methanol mixture at 80 °C for 24 h to yield green block-shaped single crystals (Supplementary Fig. 1). The as-synthesized phase (1α) has a 2D layered structure composed of one-dimensional (1D) networks of Ni paddlewheel dimers linked by the VTTF ligand, which is further pillared by 4,4’-bipyridine, based on single crystal X-ray diffraction (SC-XRD) analysis (Fig. 2a, b and Supplementary Fig. 2). The 1α phase crystallizes in the monoclinic C2/m space group, with 49% guest accessible channels occupied by solvent molecules (Supplementary Table 2); the void space was calculated with a probe radius of 1.2 Å and a grid spacing of 0.7 Å. The powder X-ray diffraction (PXRD) pattern changed significantly after the thermal activation of the 1α phase at 120 °C for ~12 h, indicating a structural transformation into the activated phase (1β) (Supplementary Fig. 3–5). Thermogravimetric analysis (TGA) showed that the 1β phase did not contain the solvent molecules, and the framework was stable up to ~380 °C (Supplementary Fig. 6). The activated crystals have a submicron sheet-like morphology (Supplementary Fig. 7), with sizes unsuitable for SC-XRD analysis, as shown by transmission electron microscopy (Supplementary Fig. 8). Nevertheless, images from selected area electron diffraction showed that they remain crystalline (Fig. 2d and Supplementary Fig. 9). Therefore, to elucidate the structure of the 1β phase, we used an advanced characterization technique and the three-dimensional electron diffraction (3D ED) method. The 3D ED analysis showed that the 1β crystallizes in the triclinic space group (*P–1*) with a huge deformation in the framework structure (Supplementary Table 3). The VTTF and 4,4’-bipyridine connectivity to the Ni paddlewheel dimer was maintained but was significantly distorted (Supplementary Fig. 10–S12). The guest accessible space was reduced to 12%, and channel dimensions were limited to 0D discrete void cavities rather than the 2D channels observed in the 1α phase (Fig. 2c and Supplementary Fig. 10). Each of the 2D layered networks was significantly compressed, reducing the interlayer distance to 13.2 Å compared to 16.5 Å of the 1α phase. Furthermore, we conducted cyclic voltammetry (CV) measurements using 0.1 M n-Bu_4_NPF_6_ in CH_3_CN as a supporting electrolyte by dispersing PCP on top of the electrode (drop casting method) to understand the redox behavior of the PCP. The CV showed a pair of reversible redox peaks and a calculated bandgap similar to the VTTF ligand, thereby indicating that the redox property of the PCP originated from the ligand (Supplementary Fig. 13–S14).

### Sorption studies

To investigate the structural flexibility of [Ni_2_(VTTF)_2_(4,4’-bipyridine)]_n_, we first tested C_6_H_6_ as a probe guest^31–33^. When we measured C_6_H_6_ vapor sorption on 1β at 298 K, a gate-open type sorption behavior was observed with an abrupt rise in the uptake capacity at 9.5 kPa (1β to 1α⊃C_6_H_6_). Finally, at 12.6 kPa, the total adsorption capacity reached up to 120 cm^3^/g, which is equivalent to 3 C_6_H_6_ molecules per Ni (1α⊃3C_6_H_6_) per asymmetric unit or Ni center (Supplementary Fig. 15). Consistent with the sorption result, the SC-XRD analysis of benzene-soaked crystals showed four adsorbed C_6_H_6_ sites per Ni, indicating strong host-guest interactions (Supplementary Fig. 16–17).

**Fig. 3:**
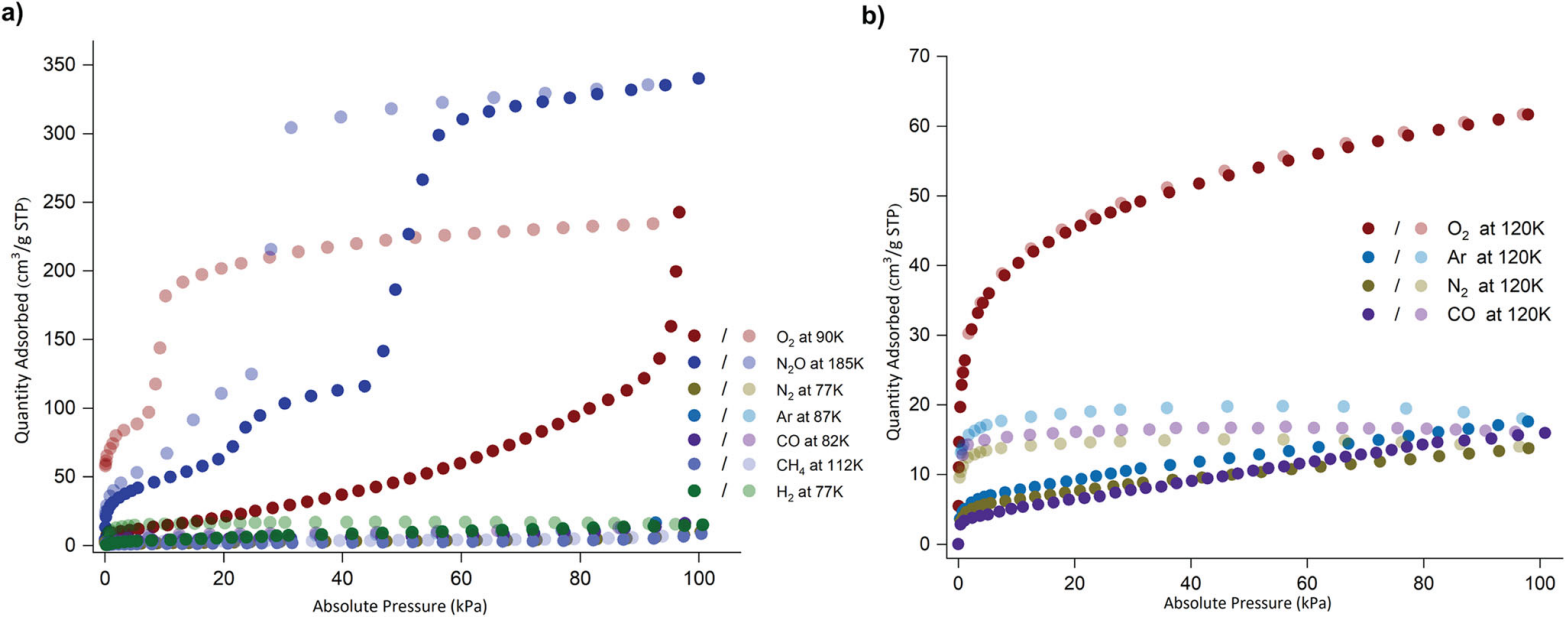
Various gas sorption measurements were conducted up to 1 bar. **a** Sorption of O_2_ (90 K), N_2_O (185 K), N_2_ (77 K), Ar (87 K), CO (82 K), CH_4_ (112 K), and H_2_ (77 K). **b** Sorption of O_2_, N_2_, Ar, and CO were measured at 120 K.

The gas sorption behaviors of [Ni_2_(VTTF)_2_(4,4’-bipyridine)]_n_ were further evaluated considering the redox properties and structural transformations induced using DMF or benzene. Up to saturated pressure, 1β showed no sorption toward N_2_ (77 K) and Ar (87 K) (Fig. 3a). However, a sizable O_2_ sorption capacity was observed at 90 K with a gradual increase in the uptake with pressure, accompanied by the gate-open behavior at 95 kPa, finally reaching 250 cm^3^ g^−1^ at ~100 kpa (Fig. 3a). The desorption profile showed significant hysteresis, with two steps with sudden drops at 10 and 5 kPa. This observation prompted us to probe the sorption of the other gases, for example, CO, CH_4_, H_2_, and N_2_O (Fig. 3a). Upon exposure to CO (82 K), CH_4_ (112 K), and H_2_ (77 K), the 1β⊃empty does not show a strong affinity with them, implying that the framework is only selective for electron-withdrawing species. Such O_2_ selective gate-open behavior was rarely been observed, except for [Zn(TCNQ–TCNQ)bpy]^30^.

To examine other electron-withdrawing gases, we measured the gas sorption isotherms for N_2_O (185 K) and CO_2_ (195 K) (Fig. 3a and Supplementary Fig. 18). The N_2_O sorption profile showed a two-step gate opening with a much higher total uptake than any other gases. Up to 20 kPa, a first plateau reaches 60 cm^3^/g, and a further increase in the pressure results in a second gate opening with an uptake capacity of 120 cm^3^/g. A sudden step at 42 kPa occurs following an increase in the uptake (300 cm^3^/g) before 60 kPa. Finally, uptake reached saturation level (350 cm^3^/g) with a gradual increase in pressure up to ~100 kPa, which is similar to the NO uptake in [Zn(TCNQ–TCNQ)bpy]^30^. Furthermore, CO_2_ sorption at 195 K (Supplementary Fig. 18) exhibits S-shaped sorption with uptake limited to only 120 cm^3^ g^−1^ at ~100 kPa. The CO_2_ uptake at ~100 kPa was consistent with the uptake observed before the second gate opening for N_2_O. These measurements show that the framework has a stronger affinity for N_2_O than for CO_2_. These observations could be due to the favorable interaction between the electron-donating framework and the electron-withdrawing guest species. As discussed above, the previously studied flexible PCPs are limited to sorption at cryogenic temperatures. We tested O_2_, Ar, N_2_, and CO gas sorption measurements from 120 to 200 K at 10 K intervals to challenge the most difficult separation of gases above their boiling points (Fig. 3b and Supplementary Figs. 19–23). The best selectivity of O_2_ was obtained at 120 K. A typical type-I sorption profile was observed for O_2_ with an uptake amount reaching 60 cm^3^ g^−1^ at ~100 kPa and desorption following the adsorption isotherm. In contrast, Ar, N_2_, and CO sorption uptakes were limited to only 15 cm^3^ g^−1^ (Fig. 3b).

To address the gas mixture selectivity, we used ideal-adsorbed solution theory (IAST) selectivity^34^. IAST selectivities at 120 K sorption were found to be 809 and 445 for O_2_/N_2_ mixtures at 1:1 and 1:9 ratio, respectively. High selectivity of 158 was also observed for O_2_ over Ar at 1:1 and 1:9 mixtures (Supplementary Fig. 24). To calculate Q_st_^35^, we used sorption isotherms measured at two temperatures at 10 °C intervals. The estimated Q_st_ showed that the 1β phase has a higher interaction energy for O_2_ (15.5 kJ/mol) than N_2_ (13.6 kJ/mol) and Ar (13.06 kJ/mol) at full loadings (Supplementary Fig. 25), confirming that 1β exhibits stronger host-guest interactions with O_2_.

### In-situ sorption studies

**Fig. 4:**
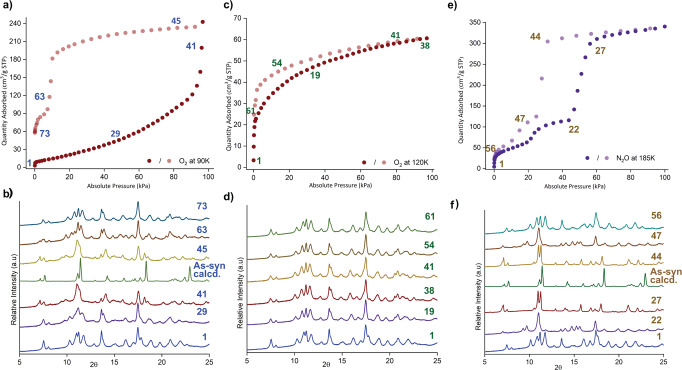
In-situ PXRD measurements were measured upon adsorption and desorption. **a**, **b** Several selected PXRD patterns were plotted for O_2_ sorption at 90 K, showing reversible 1β⊃empty to 1α⊃O_2_ transformations. **c**, **d** similarly, in-situ PXRD upon O_2_ sorption at 120 K indicates that 1β⊃empty to 1β⊃O_2_ with some shift in the peaks, corresponds to structural distortion in linkers (Supplementary Fig. 30). **e**, **f** N_2_O sorption induces two-step gate opening, 1β⊃empty to intermediate (1γ⊃N_2_O) and then to 1α⊃N_2_O phase in a reversible manner.

To gain insight into the selective sorption and the accompanying structural transformations, we performed in-situ PXRD upon every adsorption and desorption point (Fig. 4). The in-situ PXRD patterns upon O_2_ adsorption at 90 K showed that the structure does not change below 40 kPa (Fig. 4a and Supplementary Fig. 26).

The PXRD patterns showed a gradual shift toward the smaller angle above 40 kPa. With the sharp increase in the O_2_ uptake at 95 kPa, the PXRD pattern transformed into a pattern similar to the 1α phase (Fig. 4a and Supplementary Fig. 27). During desorption, the 1α⊃O_2_ phase was maintained up to 20 kPa before suddenly transforming into the 1β phase at 10 kPa. In the case of the O_2_ sorption at 120 K, the 1β⊃O_2_ does not undergo significant structural changes; instead, some PXRD patterns shifted toward lower angles, indicating the pore expansion due to the O_2_ sorption (Fig. 4c, d, Supplementary Figs. 28–29). In-situ PXRD studies showed that it has two distinct structural transformations upon N_2_O sorption (Fig. 4e, f and Supplementary Figs. 30–31). At 20 kPa, the phase transformation from 1β⊃N_2_O to intermediate (1γ⊃N_2_O) phases was observed (Fig. 4e, f and Supplementary Fig. 30). Then, at 42 kPa_,_ a sudden structural transition occurred from 1γ⊃N_2_O to 1α⊃N_2_O phase occurred. The PXRD pattern at the fully loaded (1α⊃N_2_O) phase showed the framework’s expansion compared to the as-synthesized crystal structure, which could attributed to an increased uptake of electron-withdrawing N_2_O guests (Supplementary Figs. 31–32).

**Fig. 5:**
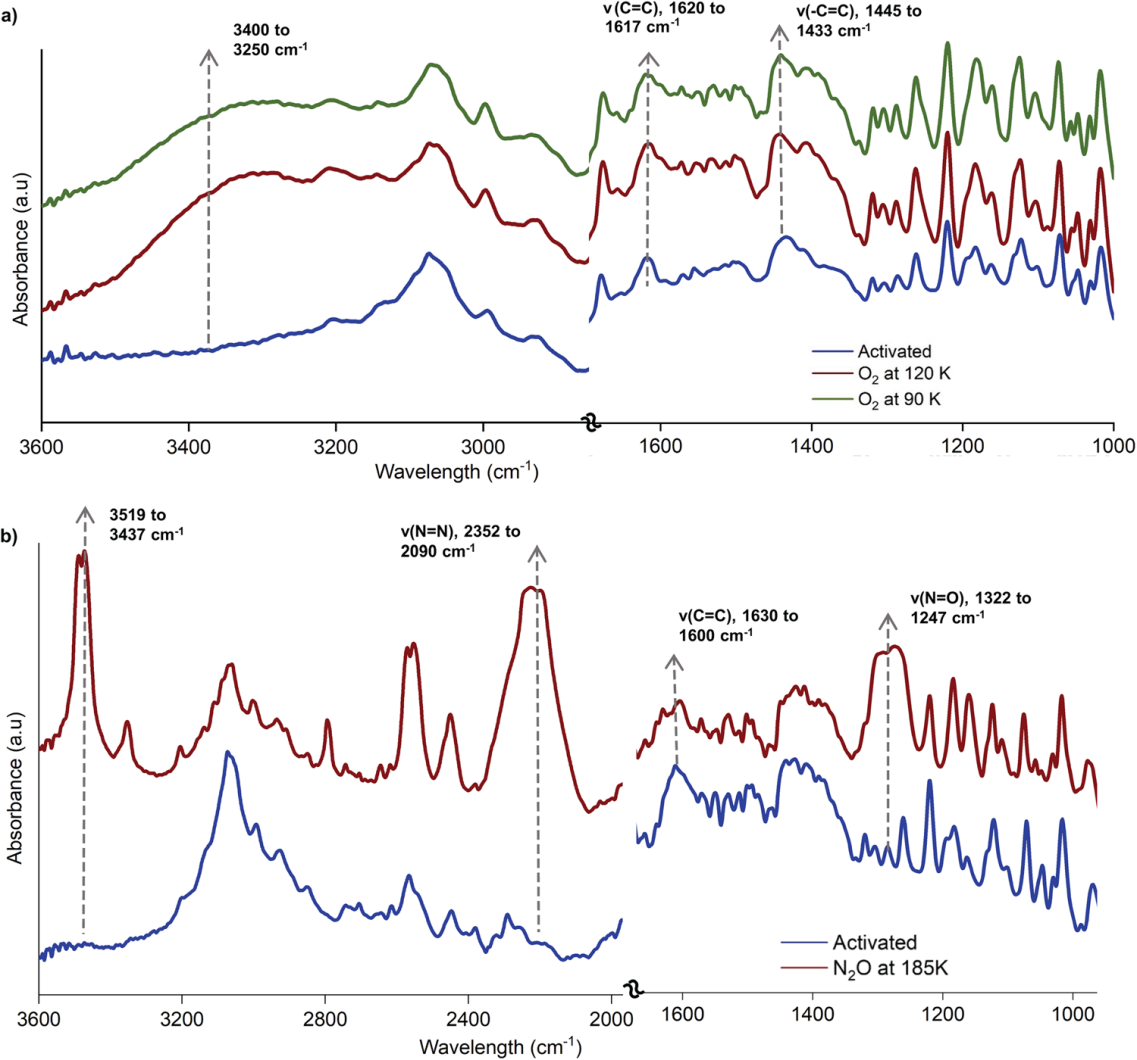
In-situ FT-IR spectra. **a** In-situ FT-IR data were measured upon O_2_ (**a**) and N_2_O adsorption (**b**).

To demonstrate the effectiveness of the donor ligand in the selective sorption property, we used in-situ optical spectroscopy measurements. In-situ Fourier-transform infrared spectroscopy (FT-IR) was used as a probe to monitor the guest species’ vibrational modes and the host framework’s functional groups upon gas adsorption (Fig. 5 and Supplementary Figs. 33–34). FT-IR spectra show a significant shift in the ν(C=C) alkene bonds at 1445 cm^−1^ during O_2_ loading at 120 and 90 K, as well as the appearance of a broad peak at around 3300 cm^−1^^36^, indicating guest inclusion and strong host-guest contacts induced at a specific region. In the presence of N_2_O at 185 K, prominent bands appeared at 2352–2090 cm^−1^ (ν(N=N)) and 1322–1247 cm^−1^ (ν(N=O), corresponding to the stretching bands of N_2_O. In addition, the obvious peak shifts of ν(C=C) at 1600–1630 cm^−1^ were observed, which is attributed to the strong host-guest interactions among N_2_O and the framework. Another broader frequency band found at a higher range (3519–3437 cm^−1^) would be attributed to the gas phase N_2_O molecule^37^.

### Theoretical calculations

To better understand the preferential adsorption of electron-withdrawing gas molecules over others, we carried out density functional theory (DFT) calculations on the binding energies of several gas species (Ar, N_2_, O_2_, and N_2_O) with the pore surface of 1β. The adsorption structures were obtained on the basis of classical Monte Carlo simulations, followed by geometry optimization using the periodic density DFT method with the PBE-D3 functional (see Materials and Methods and Supplementary Information for computational details). The calculated adsorption positions of these gas species are similar (Supplementary Fig. 35), indicating four different plausible adsorption sites, consistent with the experimental adsorption amount (~60 cm^3^/g) in 1β. Gas molecules adsorbed at these sites are close to the vinylogous tetrathiafulvalene moiety with similar orientation and interact with the framework via non-covalent interaction (Supplementary Fig. 36). As expected, the binding energy (BE) for the adsorption of different gas molecules decreases in the order of BE(N_2_O) > BE(O_2_) > BE(N_2_) > BE(Ar) for all sites (Table 1) (the negative value of BE means adsorption is exothermic); this BE order is consistent with the experimental results that this PCP can selectively adsorb O_2_ over Ar. The BE was decomposed into the framework deformation energy (*E*_def_) of PCP induced by gas adsorption and the interaction energy (*E*_int_) between gas molecules and the PCP framework. The *E*_def_ term is small and similar for the adsorption of different gas molecules (Table 1), whereas the *E*_int_ term decreases in the order of N_2_O > O_2_ > N_2_ > Ar, which is consistent with the BE order, indicating that the trend in BE is mainly determined by the interaction energy between gas molecule and PCP. To better understand the role of redox ligand in stabilizing O_2_ adsorption, energy decomposition analysis was performed on the interaction energy between gas molecule and several fragments around the adsorbed gas molecule at site I. Although the dispersion energy (*E*_DIS_) contributes the most to the total interaction energy (*E*_PBE-D3_), they are very similar for Ar, N_2,_ and O_2_ adsorptions, as shown in Supplementary Table 4, indicating that *E*_DIS_ is not the cause of the selective adsorption of O_2_ in this PCP. Similarly, the electrostatic interaction (*E*_ES_) and exchange repulsion (*E*_EXR_) terms are also very similar. However, the charge transfer, polarization and mixing term (*E*_CT+Pol+Mix_) is much larger in O_2_ case than in Ar and N_2_ cases. Particularly, when these gas molecules interact with the S-containing electron donor linkers, the difference in the *E*_CT+Pol+Mix_ term becomes larger (Fragments 1 and 2). Therefore, the electron donor linkers enhance the *E*_CT+Pol+Mix_ term for O_2_ adsorption, resulting in a more negative BE compared with Ar and N_2_ adsorptions. The Bader charge analysis shows that charge transfer occurs from the PCP framework to gas molecules and decreases in the order O_2_ (−0.13e) < N_2_O (−0.04e) < N_2_ (−0.03e) < Ar (−0.01e), which is consistent with the larger *E*_CT+Pol+Mix_ term for O_2_ adsorption.

**Table 1:** The obtained binding energy (BE), deformation energy (E_def_), and interaction energy (E_int_) from the calculations.

	Ar			N_2_			O_2_			N_2_O		
	BE	*E*_def_	*E*_int_	BE	*E*_def_	*E*_int_	BE	*E*_def_	*E*_int_	BE	*E*_def_	*E*_int_
Site I	−2.5	1.2	−3.7	−3.3	1.3	−4.7	–4.7	0.8	−5.4	−6.2	1.3	−7.6
Site II	−2.2	1.3	−3.5	−3.5	1.6	−5.1	–4.0	1.1	−5.1	−7.8	1.4	−9.2
Site III	−0.9	2.2	−3.1	−1.8	2.3	–4.1	–3.2	1.3	−4.5	−5.2	2.2	−7.4
Site IV	−2.1	1.7	−3.8	−2.8	1.7	−4.5	–4.7	0.8	−5.5	−4.0	2.2	−6.2

PBE-D3-calculated BE (kcal mol^−1^) of one gas molecule (G, G = Ar, N_2_, O_2_, and N_2_O) with [Ni_2_(4,4′-bipyridine)(VTTF)_2_]n and the corresponding E_def_ of framework and E_int_ between gas molecule and [Ni_2_(4,4′-bipyridine)(VTTF)_2_]n at different adsorption sites.

As the PCP adsorbs O_2_ or N_2_O, it transforms from the close (1β) phase to the open (1α) phase. We calculated the BE of O_2_ and N_2_O in the (1α) phase for three different binding sites and compared them with the BE in the (1β) phase. These binding sites are depicted in Supplementary Fig. S37, where we use Arabic numerals for binding sites in the open (1α) phase (Roman numerals were used for sites in the close, 1β, phase). In site 1, the gas molecule sits in a pore defined by several 6-membered carbon rings of the VTTF linkers (Supplementary Fig. 37a). In site 2, the gas molecule sits in a pore defined by 6-membered carbon rings and sulfur-containing five-membered rings of the VTTF linkers (Supplementary Fig. 37b). In site 3, the gas molecule sits in a pore defined by the 4,4’-bipyridine linker as well as the 6-membered carbon rings and sulfur-containing five-membered rings of the VTTF linkers (Supplementary Fig. 37c).

We find that the BE/*E*_def_/*E*_int_ values for O_2_ in sites 1, 2, and 3 in the (1α) phase are −3.0/0.3/−3.3, −4.3/0.1/−4.3, and −5.1/0.1/−5.2 kcal mol^−1^ respectively. For N_2_O, the BE/*E*_def_/*E*_int_ values in sites 1, 2, and 3 in the (1α) phase are −6.3/0.3/−6.6, −6.6/0.4/−7.0, and −7.0/0.2/−7.2 kcal mol^−1^, respectively. We see for both gases that the binding energy is highest in site 3, followed by site 2, and then site 1. We conjecture that this trend occurs due to stronger interactions between the gas molecule and the polarizable framework sulphur atoms in sites 2 and 3 and framework nitrogen atoms in site 3. Site 3 is also a smaller pore which allows for framework atoms to be in closer proximity to the gas molecule. Compared to sites 1–4 in the (1β) phase, the BEs are similar, however, the deformation energies (*E*_def_) and interaction energies (*E*_int_) are smaller in magnitude. This is consistent with the adsorbed molecule being bound in the larger pores of the open phase of this PCP, with fewer framework atoms closely enclosing the adsorbed molecule, reducing *E*_int_, and thus producing less framework deformation, reducing *E*_def_, relative to the close phase.

To investigate the gate-opening process of this PCP upon adsorption of N_2_O, we performed DFT calculations of the zero-temperature formation energy of the (1α) and (1β) phases, when empty, and when containing 0.5, 5.5, and 11 adsorbates per framework Ni atom. When empty, the formation energy of the (1β) phase is smaller than that of the (1α) by 20.5 kcal mol^−1^ per Ni framework atom. When filled with 0.5 N_2_O for every Ni framework atom, the (1β) phase is even more strongly favored because the N_2_O can bind in site II of the (1β) phase (−7.8 kcal mol^−1^ BE) and site 3 of the (1α) phase (−7.0 kcal mol^−1^ BE). So the difference in zero-temperature formation energy of the (1α) and (1β) phases with 0.5 N_2_O loading per Ni framework atom is 20.9 kcal mol^−1^ per Ni atom in favor of the (1β) phase. The situation changes upon a loading of 5.5 adsorbates per Ni framework atom and the formation energy of the (1β) phase is now only 3.8 kcal mol^−1^ lower than the (1α) phase. At this loading the per adsorbate average binding energy is −3.5 kcal mol^−1^ for the (1β) phase and −6.5 kcal mol^−1^ for the (1α) phase, as might be expected for a loading of the (1β) phase that is near its maximum capacity, forcing N_2_O in to unfavorable constrained binding locations. Because the difference in marginal adsorbate BE is likely greater, we assume the loaded (1α) phase to have a lower formation energy of 6.5 adsorbates per Ni atom. This loading range, around 190 cm^3^ g^−1^ (6 adsorbates per Ni atom), falls within the phase transition region shown in Fig. 4c. We loaded 11 N_2_O molecules per Ni atom into the (1α) phase, corresponding to 350 cm^3^ g^−1^, whereas this loading was impossible in the (1β) phase.

## Conclusions

In summary, we present a 2D flexible porous coordination network, Ni_2_(4,4’-bipyridine)(VTTF)_2_ that exhibits a structural transformation upon the selective sorption of O_2_ and N_2_O. Single crystal, electron diffraction studies, in-situ PXRD, and FT-IR measurements were used to elucidate the origin of structural flexibility and the transformation mechanism. Furthermore, modeling studies provide additional insights into the O_2_ and N_2_O binding sites and the origin of selective sorption; our calculations show that the selectivity originates from the electron transfer functionality and flexibility of PCP. Importantly, we show that frameworks constructed from suitable metals and linkers with electron donor or acceptor functionality, coupled with framework flexibility, could be potential candidates for differentiating guest species that have similar physicochemical properties.

In this study, we demonstrated that porous materials with flexibility and electron donor functionality, are promising avenues for the selective adsorption of electron-withdrawing guest species. Alternative strategies have been proposed, but they generally require high regeneration energy, display-poor selectivity, or rely solely on flexibility, which does not necessarily translate into a high separation efficiency for gases with different gate-opening pressures. A future direction would be to combine different strategies to increase the separation efficiency, for example, the design of flexible frameworks possessing interactions involving charge and spin for magnetically active guest species, and distinguish non-magnetically active species. This could enable efficient separation of paramagnetic (O_2_) from diamagnetic molecules, such as N_2_ and Ar. This in turn could enable the design of the next generation of porous materials for low-energy and cost-efficient O_2_ selective separation from the air, which remains one of the most significant challenges in current gas separation technologies.

## Materials and methods

### Synthesis of [Ni_2_(4,4′-bipyridine) (VTTF)_2_] (VTTF = 2,2′-[1,2-bis(4-benzoicacid)−1,2ethanediylidene]bis-1,3-benzodithiole) • DMF (as-synthesized or 1α)

A mixture of Ni(NO_3_)_2_·6H_2_O (14.5 mg, 0.05 mmol; purchased from Sigma-Aldrich), 4,4’-bipyridine (8 mg, 0.05 mmol; purchased from Sigma-Aldrich), H_2_VTTF, (23.5 mg, 0.05 mmol; prepared as previously reported procedure^38^) in DMF (7.5 mL)/MeOH (2.5 mL) was added to a 20 mL glass vial. The vial was capped tightly and placed in an oven at 80 °C for 48 h to yield block-shaped green crystals. The contents of the vial were allowed to cool at room temperature. Crystals were filtered and washed with 5 mL of DMF for three times. Calculated yield was found to be >60%.

### Preparation of [Ni_2_(4,4′-bipyridine) (VTTF)_2_] (Activated or 1β)

As synthesized sample were heated at 120 °C for ~12 h under vacuum or solvent exchange with methanol (three times/day) for 3 days followed by heating at 80 °C for 12 h under vacuum.

### TGA, PXRD and SCXRD

The TGA measured from a Rigaku TG 8120 analyser (EVO2 TG/S-SL) using a heating rate of 10 °C min^−1^ under N_2_ flow. Powder X-ray diffraction (PXRD) data were recorded with Rigaku Smart lab X-ray diffractometer with 2D array detector using Cu Kα radiation (*λ* = 1.54178 Å). Single-crystal diffraction data were collected on a Bruker Quest diffractometer equipped with a CMOS detector and IμS microfocus X-ray source (Cu K_α_, *λ* = 1.5418 Å). Absorption corrections were applied by using the multi-scan program SADABS. Indexing was performed using APEX2 [Bruker, 2012] (Difference Vectors method). Data integration and reduction were performed using Saint Plus [Bruker, 2012]. Space group was determined using XPREP implemented in APEX2 [Bruker, 2012]. Structural solution and refinement against *F*^*2*^ were carried out using the SHELXL programs.

### Gas and vapor sorption

Gas sorption measurements of O_2_, N_2_, Ar, CO, N_2_O, H_2_, CH_4_ and CO_2_ at different temperatures were carried out using BEL-mini, BEL-max and BEL-18 (Microtrac BEL Corp., Japan). C_6_H_6_ vapor sorption were measured on Belmax2 (Microtrac BEL Corp., Japan). Before all gas sorption and vapour separation experiments, methanol exchanged samples were reactivated at 80 °C under vacuum for 2 h.

### In-situ coincidence PXRD and FT-IR experiment

In-situ coincident PXRD measurements were measured on a Rigaku Smart lab with CuKα radiation (Rigaku, Japan) which is synchronised to a BELSORP-18PLUS volumetric sorption instrument (MicrotracBEL Japan, Corp.). Helium based cryo-system was adopted to control the temperature range. In a typical preparation method, a freshly as-synthesised sample was solvent exchanged with methanol for >3 days (three time per day) and then activated at 80 °C under vacuum overnight. The activated sample of ~80 mg was transferred to sorption equipment and reactivated in-situ at 80 °C under vacuum for 2 h to make sure no moisture adsorption on surface. The O_2_ (90 and 120 K) and N_2_O (185 and 195 K) coincidence PXRD measurements were measured at each adsorption and desorption equilibrium point of sorption isotherm. Similar procedure followed to measure in-situ FT-IR spectra. FT-IR measured on JASCO FT/IR-6100 which is synchronised to a BELSORP-18PLUS volumetric sorption instrument.

### Cyclic voltammetry (CV)

The measurements were performed in n-Bu_4_NPF_6_/CH_3_CN electrolyte using a electrochemical analyser (model 660E). Argon was bubbles through solutions of 0.1 M n-Bu_4_NPF_6_ dissolved in CH_3_CN. The electrochemical cell comprised of glassy carbon working electrode, a platinum auxiliary electrode and an Ag/AgCl electrode was selected as reference electrode. 10 mg of sample was dispersed in 1 mL of MeCN by sonication to form homogenous mixture and then drop casted on the glassy carbon electrode. All potentials are reported in mV vs. Fc/Fc^+^.

### Electron diffraction crystal structure determination

Electron diffraction measurements were collected using a Rigaku Synergy-ED^39^ equipped with a Rigaku HyPix-ED detector optimised for operation in the 3D ED experimental setup. The sample consisted of flake-like crystallites with ~300 nanometer thickness. A total of 16 data sets were collected. For improved data quality, a total of 5 data sets were merged, resulting in a comprehensive data set with a resolution limit of 0.95 Å. The initial phase for the activated phase was determined by SHELXT^40^ and the structure refinement was performed by the full matrix least-squares method of SHELXL^41^. The SFAC instructions were used to input atomic scattering factors for electrons to replace those for X-rays.

### Theoretical calculations

The binding energies of several gas species (Ar, N_2_, and O_2_) with [Ni_2_(4,4’-bipyridine)(VTTF)_2_]_n_ were calculated to understand the preferential adsorption of electron-rich gas species over others. Adsorption positions of these gas species were located using canonical Monte-Carlo (MC) simulation^42^, as implemented in RASPA^43^. The Lennard-Jones (LJ) potentials were used to describe the Van der Waals interaction of gas molecules with PCP framework and the electrostatic interaction was evaluated with the Ewald summation method. The LJ parameters for PCP framework were taken from the standard universal force field (UFF)^44^ and the DDEC atomic charges^45,46^ of PCP framework were used in the evaluation of electrostatic interaction. The LJ parameters and atomic charges of Ar, N_2_, and O_2_ were taken from the TraPPE force field^47^. In the MC simulation, the first 1 × 10^5^ cycles were employed for obtaining equilibration and then 3 × 10^5^ cycles were used for obtaining distribution of guest molecule at room temperature. The final gas adsorption configuration obtained by above MC simulation was used to construct the initial structure for performing geometry optimization with density functional theory (DFT).

The binding energy for gas adsorption was calculated using spin-polarized DFT method with periodic boundary conditions as implemented in the Vienna Ab initio Simulation Package (VASP 5.4.4)^48,49^. The Perdew-Burke-Ernzerhof functional^50^ with Grimme’s semi-empirical “D3” dispersion term^51^ (PBE-D3) was employed in these calculations. The plane wave basis sets with an energy cutoff of 500 eV were used to describe valence electrons and the projector-augmented-wave pseudopotentials^52,53^ were used to describe core electrons. The threshold for atomic force convergence was set to be 0.01 eV/Å in geometry optimization. The Brillouin zone was sampled by a Γ-point in these calculations. The Ni-Ni paddlewheel unit was treated as anti-ferromagnetic and each Ni^2+^ has two unpaired electrons^54,55^. The Hubbard U correction^56^ was applied to the d electrons of Ni atoms (*U*_eff_ = 6.4)^57^. Additional details can be found in the Supplementary Information.

## Supplementary information

Supplementary Information

Description of Additional Supplementary Files

Supplementary Data 1

Supplementary Data 2

Supplementary Data 3

Supplementary Data 4

Supplementary Data 5

## Data Availability

The authors declare that the data supporting the findings of this study are available within the article and the Supplementary Information as well as from the authors upon reasonable request. The input files for the VASP calculations (INCAR and POSCAR files) can be found in Supplementary Data 1 and Data 2. Supplementary crystallographic data (Supplementary Data 3–5) for this manuscript has been deposited at the Cambridge Crystallographic Data Centre under deposition numbers CCDC 2221440, 2221441 and 2222591. These data can be obtained free of charge from http://www.ccdc.cam.ac.uk/data_request/cif.
