# Supplementary material for: Selective sorption of oxygen and nitrous oxide by an electron donor-incorporated flexible coordination network
Source: Commun Chem. 2023 Apr 4;6:62. doi: 10.1038/s42004-023-00853-1 (PMC10073098; doi:10.1038/s42004-023-00853-1)
Supplement: Supplementary file 1 — Supplementary Information [file 42004_2023_853_MOESM1_ESM.pdf]

Supplementary Information

Selective sorption of oxygen and nitrous oxide by an electron donor-incorporated flexible coordination network

Mohana Shivanna,[1] Jia-Jia Zheng,[2] Keith G. Ray,[3] Sho Ito,[4] Hirotaka Ashitani,[5] Yoshiki Kubota,[5,6] Shogo Kawaguchi,[7] Vitalie Stavila,[8] Ming-Shui Yao,[1] Takao Fujikawa,[1] Ken-ichi Otake[1]* and Susumu Kitagawa[1]*

[1]Institute for Integrated Cell-Material Sciences, Kyoto University Institute for Advanced Study, Kyoto University, Yoshida Ushinomiya-cho, Sakyo-ku, Kyoto 606-8501, Japan.

[2]Laboratory of Theoretical and Computational Nanoscience, CAS Center for Excellence in Nanoscience, National Center for Nanoscience and Technology, Chinese Academy of Sciences, No. 11 ZhongGuanCun BeiYiTiao, Beijing 100190 P. R. China.

[3]Lawrence Livermore National Laboratory, Livermore, California 94550 USA.

[4]Rigaku Corporation, 3-9-12 Matsubara-cho, Akishima, Tokyo 196-8666, Japan.

[5]Department of Physical Science, Graduate School of Science, Osaka Prefecture University, Sakai, Osaka 599-8531, Japan.

[6]Department of Physical Science, Graduate School of Science, Osaka metropolitan University, Sakai, Osaka 599-8531, Japan.

[7]Japan Synchrotron Radiation Research Institute (JASRI), SPring-8, 1-1-1 Kouto, Sayo-cho, Sayo-gun, Hyogo 679-5198, Japan.

[8]Sandia National Laboratory, Livermore, California 94550 USA.

Email: ootake.kenichi.8a@ kyoto-u.ac.jp, kitagawa@icems.kyoto-u.ac.jp

Physio-chemical properties

Supplementary Table 1. Physical and electronic parameters for various gases.

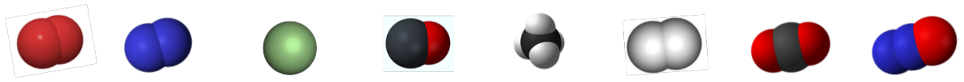

	O_2	N_2	Ar	CO	CH_4	H_2	CO_2	N_2O
Kinetic diameter(Å)	3.46	3.64	3.4	3.76	3.8	2.89	3.3	3.3
Dipole moment (D)	0	0	0	0.117	0	0	0	0.167
Quadrupole moment 10^{40} θ (cm^2)	1.3	4.7	0	8.3	0	2.21	13.4	-
Polarizability($Å^3$)	1.66	1.76	1.66	1.95	2.60	0.82	2.65	-
Boiling point (K)	92	77	87	82	112	20.28	195	185

Crystallographic details

Supplementary Table 2. Crystal data and refinement parameters for the as synthesized phase.

	**As synthesized Supplementary data 3**	**C_6H_6 inclusion structure Supplementary data 5**
Formula[a]	**$C_{70}H_{40}N_2Ni_2O_8S_8$**	**$C_{59}H_{44}NNiO_4S_4$**
cF.W.	1410.90	1017.88
T (K)	140K	140K
Space group	C*2/m*	I*2/m*
a (Å)	33.09(11)	13.583(12)
b (Å)	11.887(4)	12.073(12)
c (Å)	13.470(5)	31.179(2)
α (°)	90	90
β (°)	111.492(8)	92.511(7)
γ (°)	90	90
V (Å^3)	4930(3)	5108.5(8)
Z	2	4
D_c (g cm^{-3})	0.951	1.324
μ (mm^{-1})	0.134	0.591
Data collected/unique	5935/4089	5682/3638
R_1 (I>2σ (I))	0.0642	0.0637
wR_2 (all data)	0.2232	0.1877
Wavelength	0.71073	0.71073
GOF	1.069	1.020

Supplementary Table 3. Crystal data and refinement parameters for the activated phase from electron diffraction.

	**Activated**<br>**Supplementary data 4**
Formula[a]	$C_{35}H_{20}NNiO_4S_4$
cF.W.	705.45
T (K)	298 K
Space group	*P*-1
a (Å)	11.132(3)
b (Å)	12.874(3)
c (Å)	13.151(3)
α (°)	65.52(2)
β (°)	81.535(18)
γ (°)	88.644(19)
V (Å^3)	1695.2(7)
Z	2
No. of measured/No. of unique reflections	17842/3511
No. of parameters	503
Merged datasets	5
R_{int}/R_{pim}	0.133(0.237)[a]/0.075(0.120)[a]
R_1 [F2>2σ(F^2)]	0.2096
wR(F2) [F2>2σ(F^2)]	0.5007
2θmax(°)	1.438
Radiation type/Wavelegnth(Å)	200 keV electrons/0.0251
S	1.915
Completeness	0.993

*a Values in parentheses are for the highest resolution shell.

Crystal structural and crystal images of as-synthesized phase

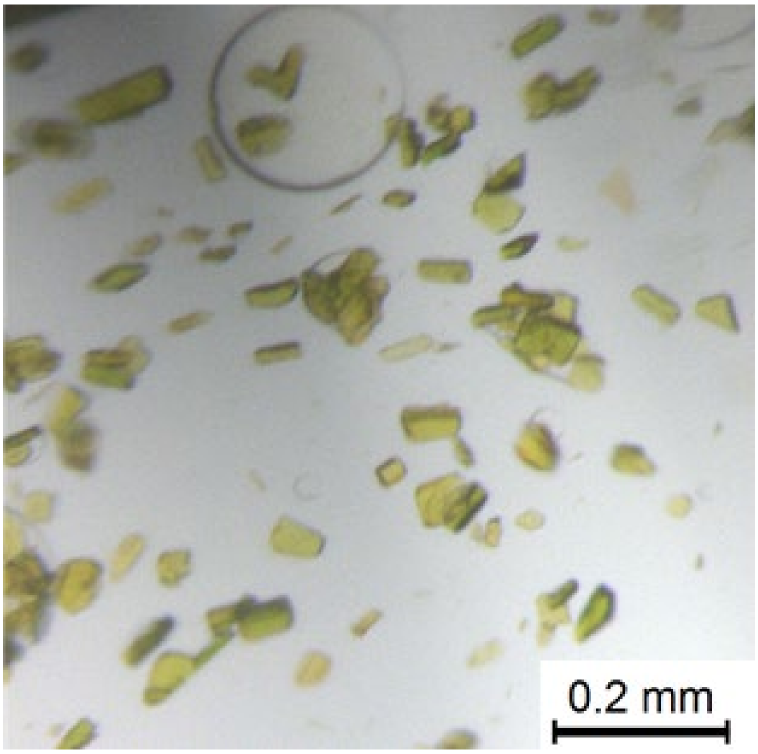

Supplementary Figure 1. Single crystal images for the as-synthesized phase.

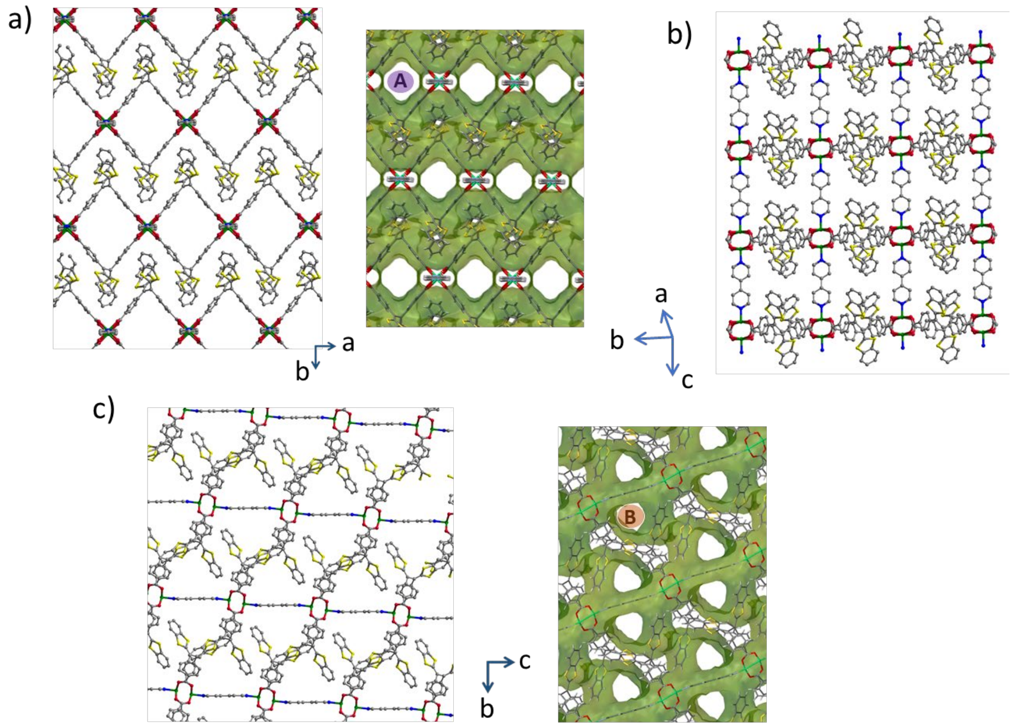

Supplementary Figure 2. Closed packed structure of α phase as shown in different directions (Green, red, blue, yellow, gray spheres represent Ni, O, N, S, and C, respectively). There are two types of cavities (A and B) formed in the channels as shown.

Comparisons of PXRD patterns

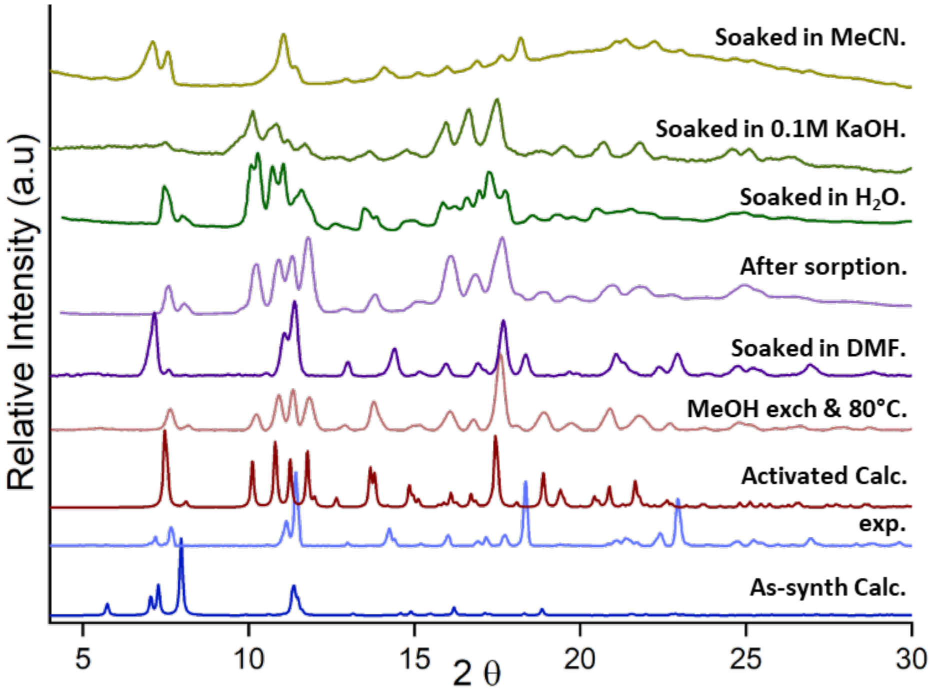

Supplementary Figure 3. Comparisons of PXRD patterns, α calc vs. exp (air dried), β calc vs. exp and after various conditions. For α phases, we observed slight difference in peak position that is due to un-stability of structure and loss of DMF lead to undergo structural transformation. Similarly, in case of β phase the shift in PXRD pattern due to difference in measurement conditions and structural flexibility.

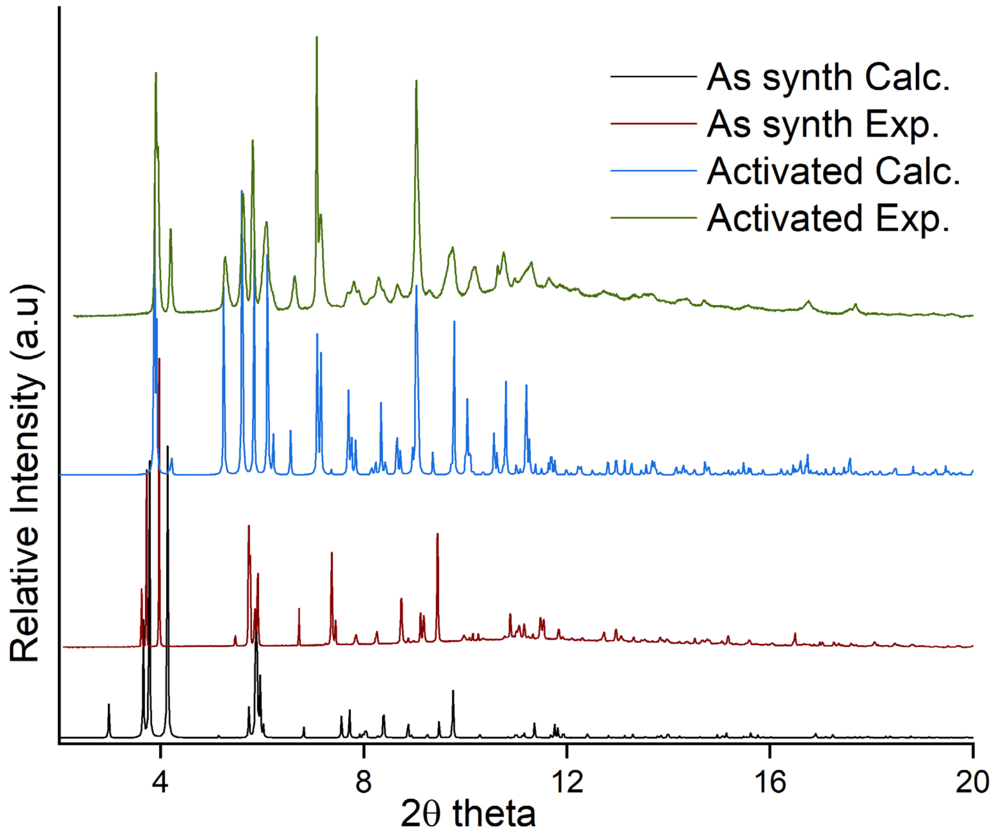

Supplementary Figure 4. PXRD patterns were measured using synchrotron diffraction with the wavelength, 0.800Å. Compared α calc (black) vs. exp (red), β calc (blue) vs. exp (green).

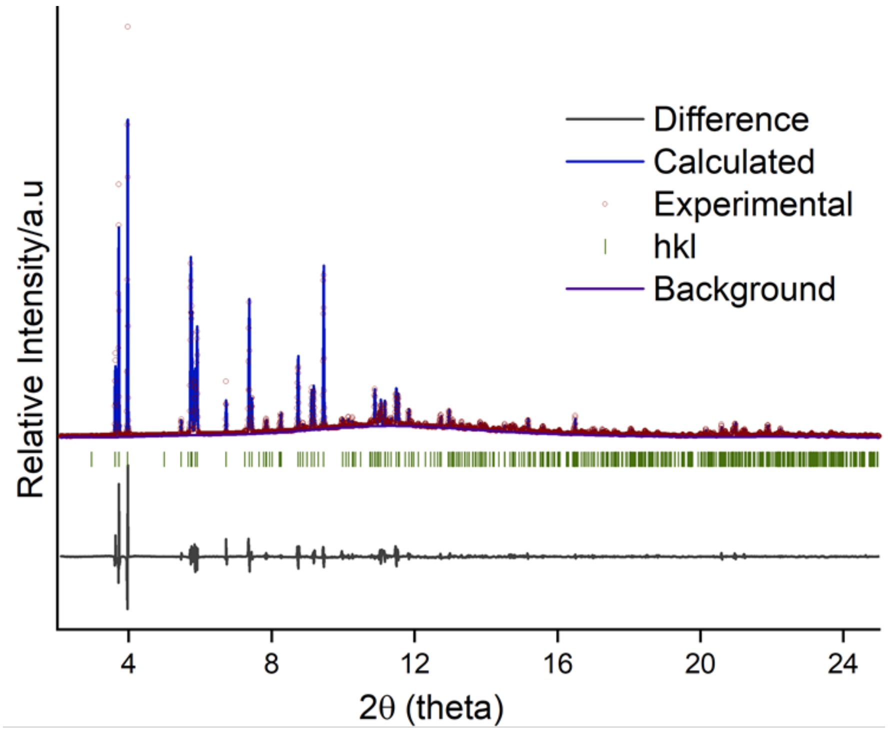

Supplementary Figure 5. The PXRD refinement results for the α phase were obtained from the Le Bail fitting. The experimental PXRD pattern (red circle) was collected using the synchrotron diffraction with the wavelength, 0.800Å. The calculated PXRD pattern (blue) obtained after refinement and the difference in diffraction patterns are represented in black. The resulted cell parameters at room temperature were slightly larger compared to as-synthesized single crystal structure (100 K). Topas software was used for the calculation. The space group and lattice parameters of as-synthesized phase (C2/m, a = 33.09(11) Å , b = 11.887(4) Å , c = 13.470(5) Å, β = 111.49(8) º) were used as the starting point for the fitting. The refinement result gave the lattice parameters of a = 33.4591(5) Å, b = 12.4457(2) Å and c = 13.6468(2) Å, β = 111.989(2)º and cell volume of 5269.4(2) $Å^3$ with Rp = 4.50% and Rwp = 7.47% (Rexp = 0.92).

TGA measurements

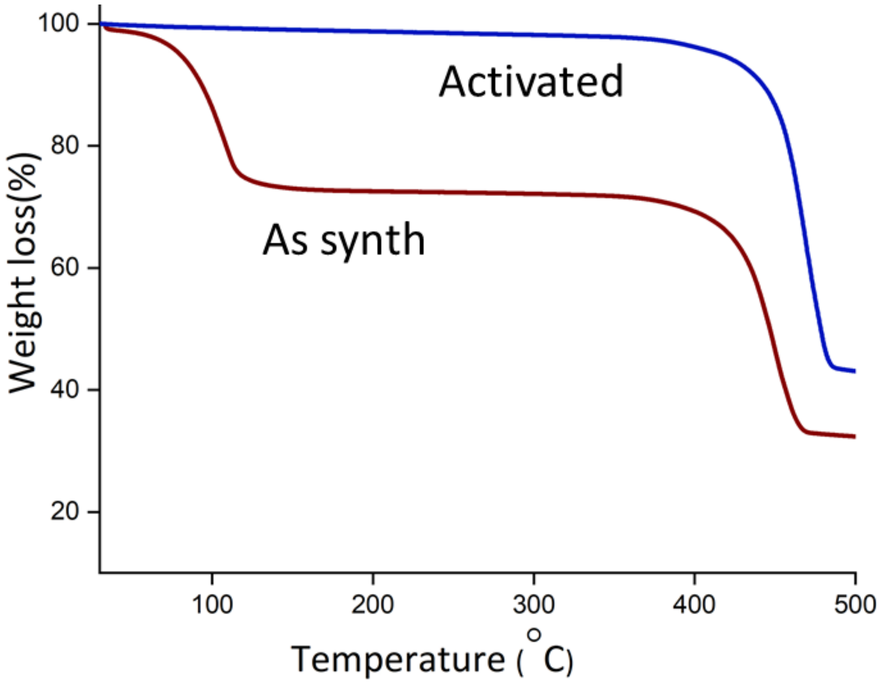

Supplementary Figure 6. TGA measurements for the as-synthesized (red) and activated phases (blue).

SEM and TEM images

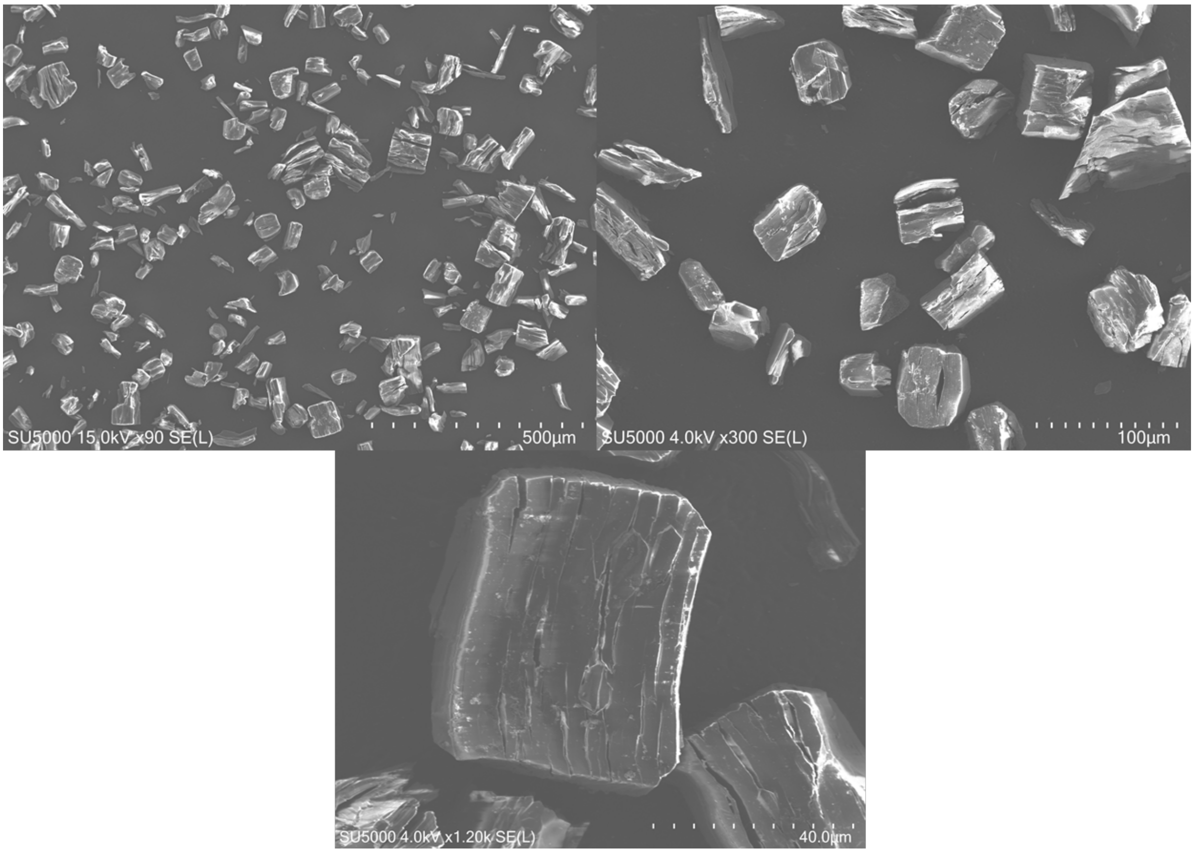

Supplementary Figure 7. SEM (scanning electron microscopic) images measured for the 1β phase.

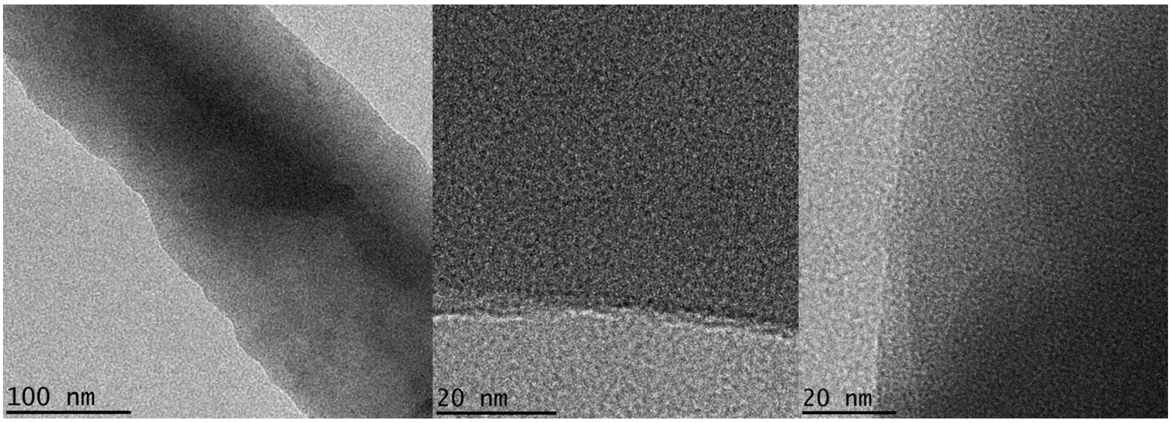

Supplementary Figure 8. The transmission electron diffraction (TEM) images measured for the 1β phase. The images were measured using JEM-2200FS configuration with the resolution 0.23nm(Point) and 0.1nm(Lattice).

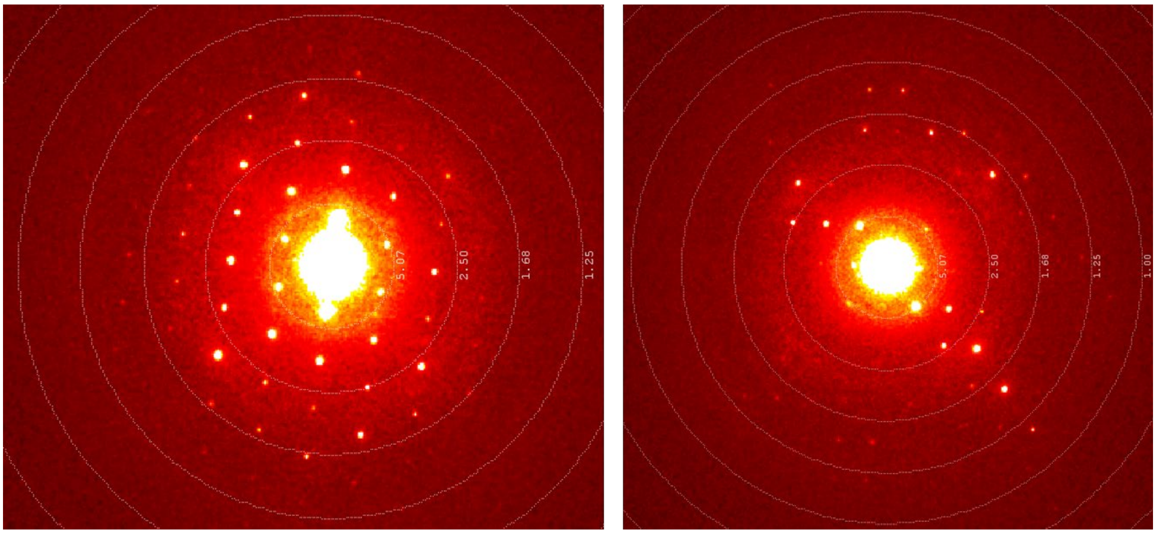

Supplementary Figure 9. The diffraction images from the electron diffraction for the 1β phase.

Structural representation of activated phase and comparison

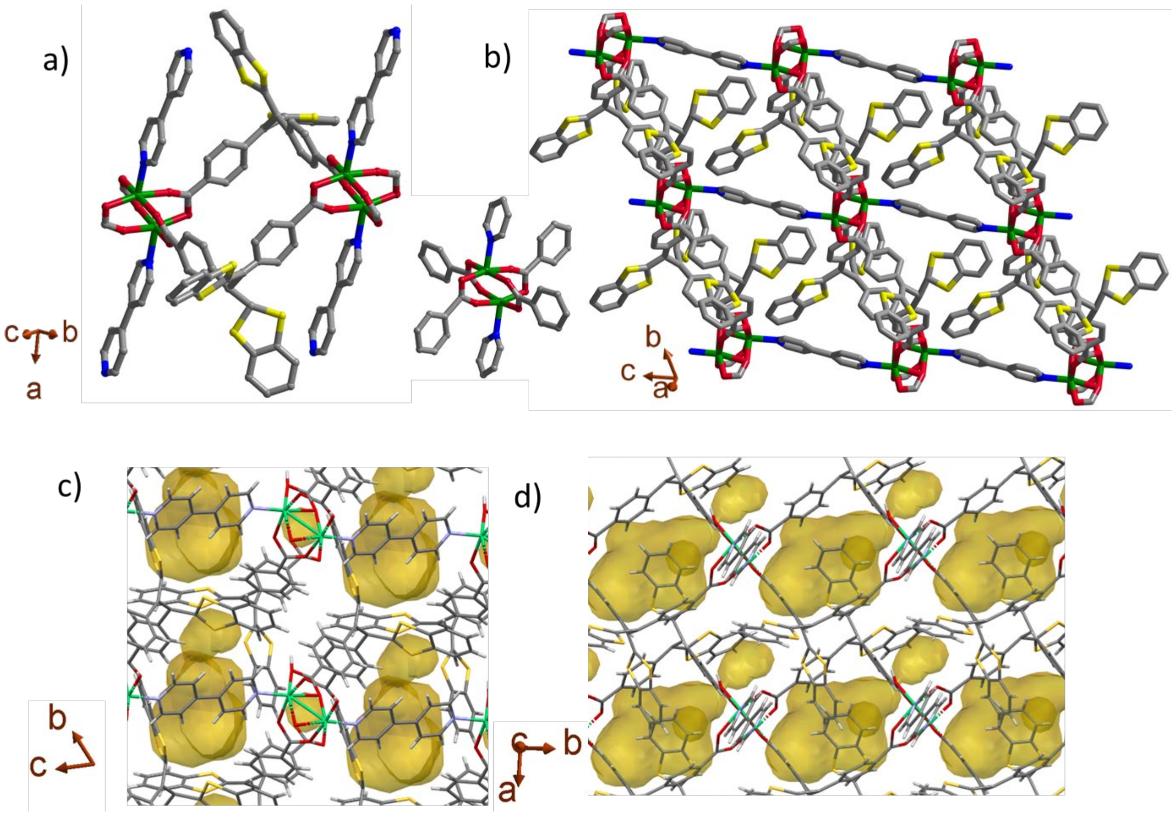

Supplementary Figure 10. Representation of 1β phase along different directions. a and b) distorted coordination unit and closed packed structure, respectively. c and d) discrete voids as shown in different directions (Green, red, blue, yellow, gray spheres represent Ni, O, N, S and C, respectively).

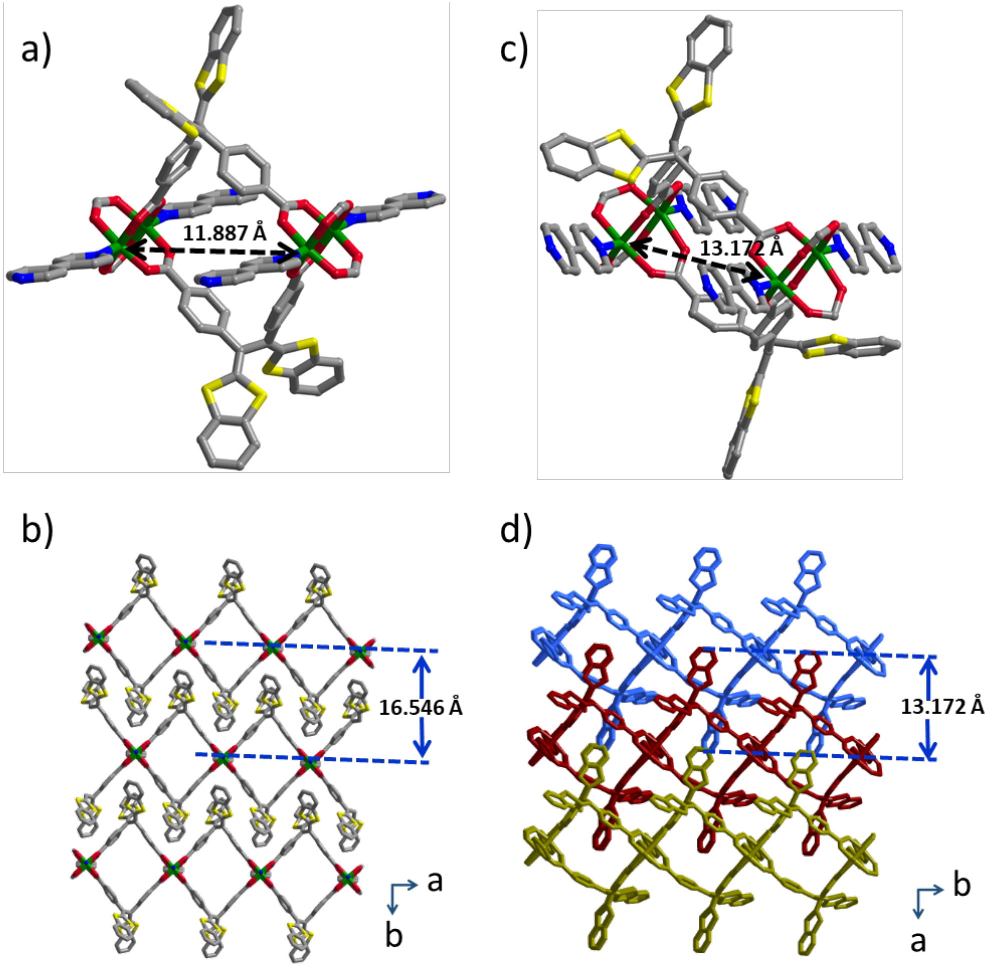

Supplementary Figure 11. a) metal-metal distance and b) interlayer distance between the 2D layers in as 1α phase. Similarly, compared for the 1β phase c) metal-metal distance and d) interlayer distance (Green, red, blue, yellow, gray spheres represent Ni, O, N, S and C, respectively).

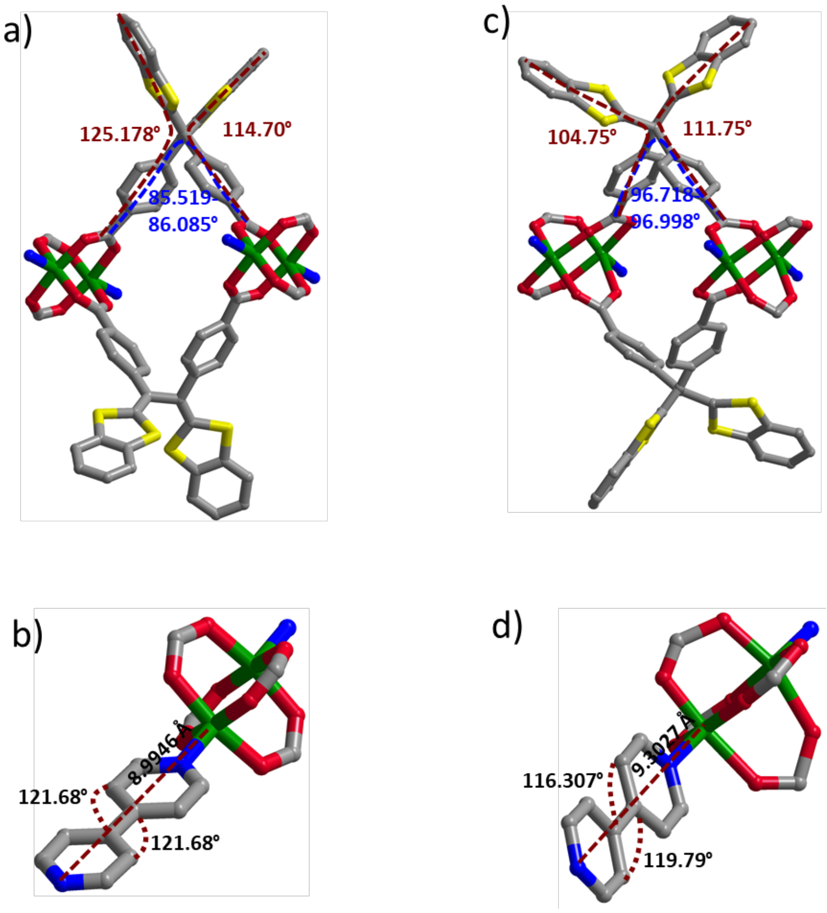

Supplementary Figure 12. VTTF and 4,4'-bipyridine bond angle distortions compared for the 1α (a and b) and 1β (c and d) phases (Green, red, blue, yellow, gray spheres represent Ni, O, N, S and C, respectively).

Cyclic voltammetry (CV)

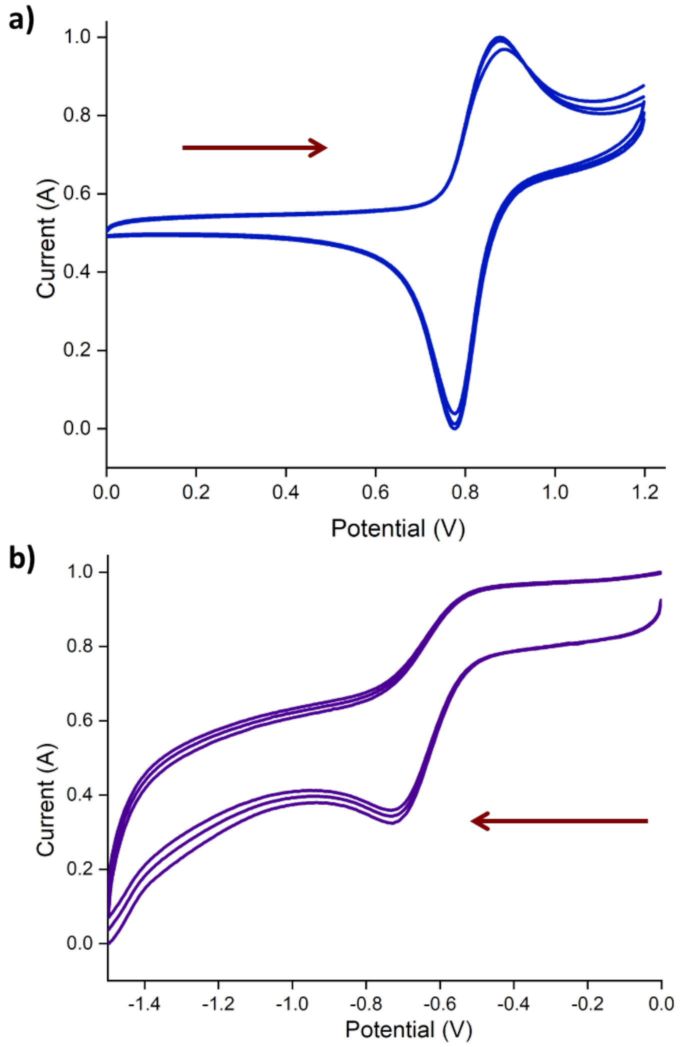

Supplementary Figure 13. Cyclic voltammetry measured for the 1β phase in liquid state. a) 0 to 1.2 V vs. current over 3 consecutive cycles (positive). Similarly, b) 0 to -1.5 V vs. current over 3 consecutive cycles (negative). All the experiments were performed in 0.1 M n-Bu_4NPF_6in CH_3CN supporting electrolyte. Arrows in a) and b) indicates the directions of forward scan. The calculated band gap was found to be 1.291 (HOMO, -4.9935 and LUMO, -3.7025).

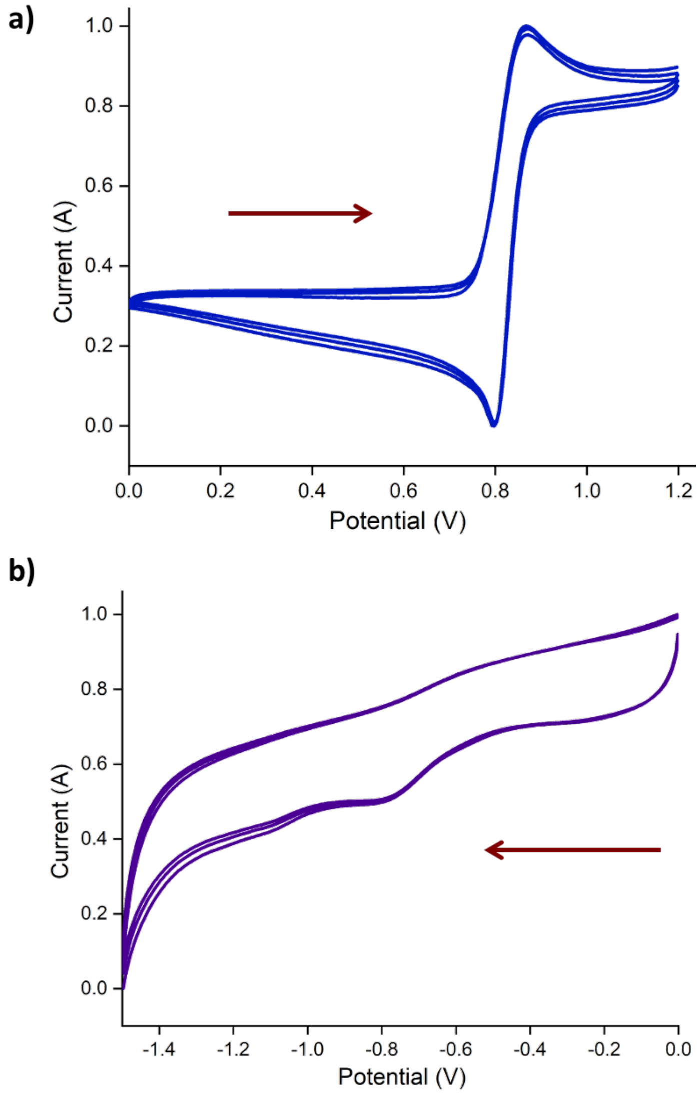

Supplementary Figure 14. Cyclic voltammetry measured for the VTTF ligand in liquid state. a) 0 to 1.2 V vs. current over 3 consecutive cycles (positive). Similarly, b) 0 to -1.5 V vs. current over 3 consecutive cycles (negative). All the experiments were performed in 0.1 M n-Bu_4NPF_6in CH_3CN supporting electrolyte. Arrows in a) and b) indicates the directions of forward scan. The calculated band gap was found to be 1.2853 (HOMO, -4.9955 and LUMO, -3.7102).

Vapor sorption

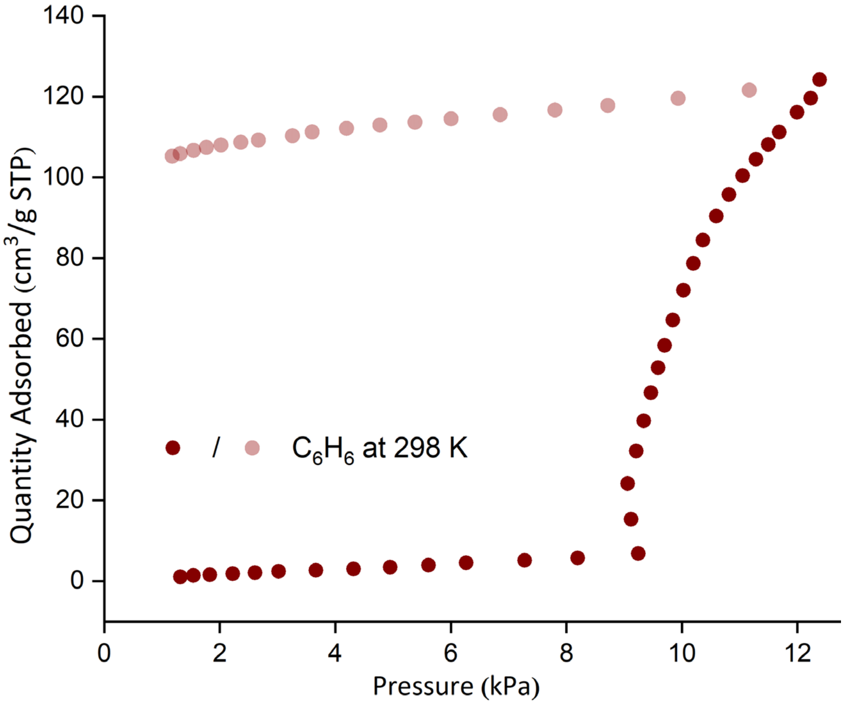

Supplementary Figure 15. Vapor sorption of benzene measured at 298 K. Benzene sorption exhibits gate opening at 9.5 kPa.

Structural representation of C_6H_6 loaded phase

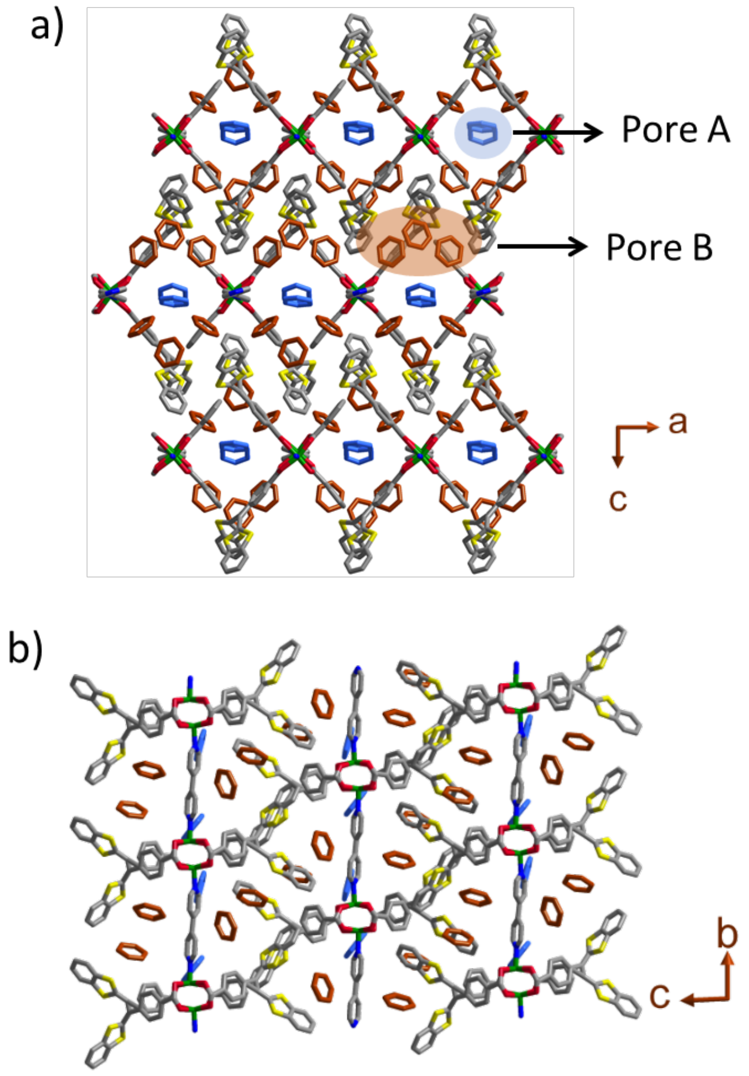

Supplementary Figure 16. Packing structure of benzene inclusion structure (1α⊃3C_6H_6) along b axis (a) and along a-axis (b). (Green, red, blue, yellow, gray spheres represent Ni, O, N, S and C, respectively).

Host-guest and guest-guest interactions

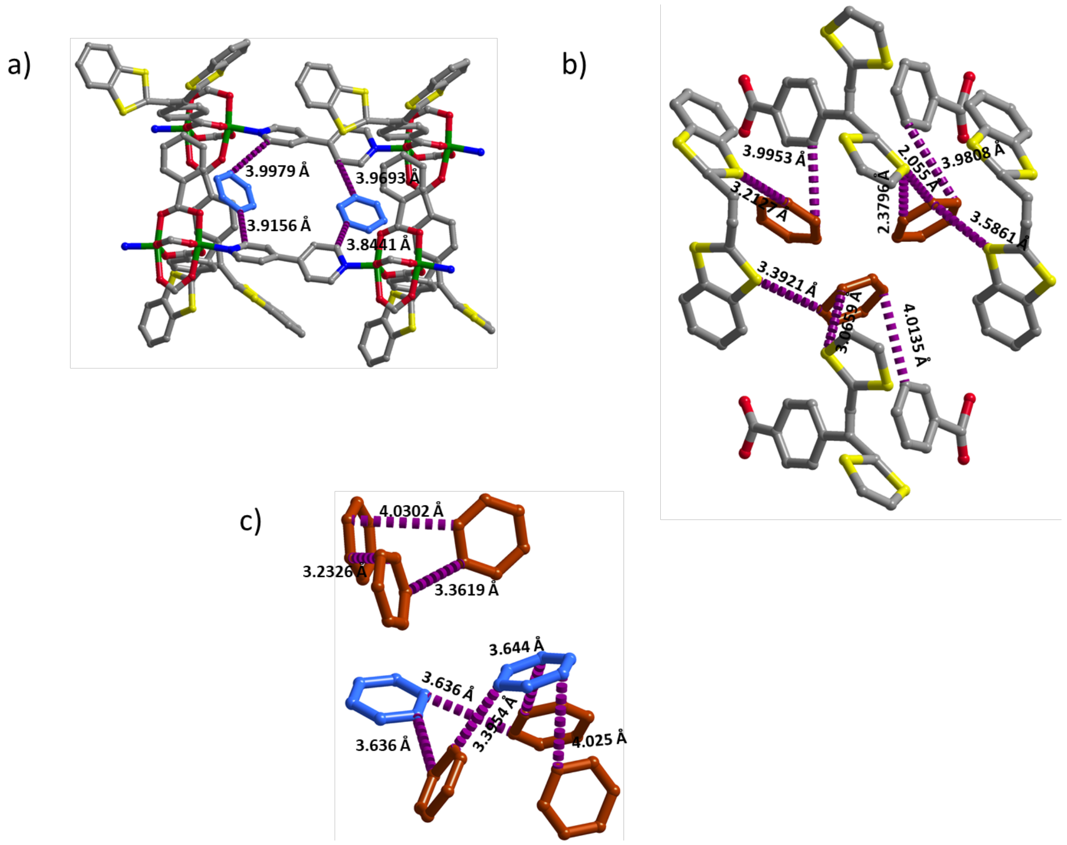

Supplementary Figure 17. Host-guest and guest-guest interactions between C_6H_6 and the framework. a) The C_6H_6 occupied at pore A forms weak host host-guest interactions. b) Strong host-guest interactions found between C_6H_6 and framwork at pore B. c) There exist strong guest-guest contacts between both C_6H_6 occupied at pore A and pore B (Green, red, blue, yellow, gray spheres represent Ni, O, N, S and C, respectively).

Sorption measurements

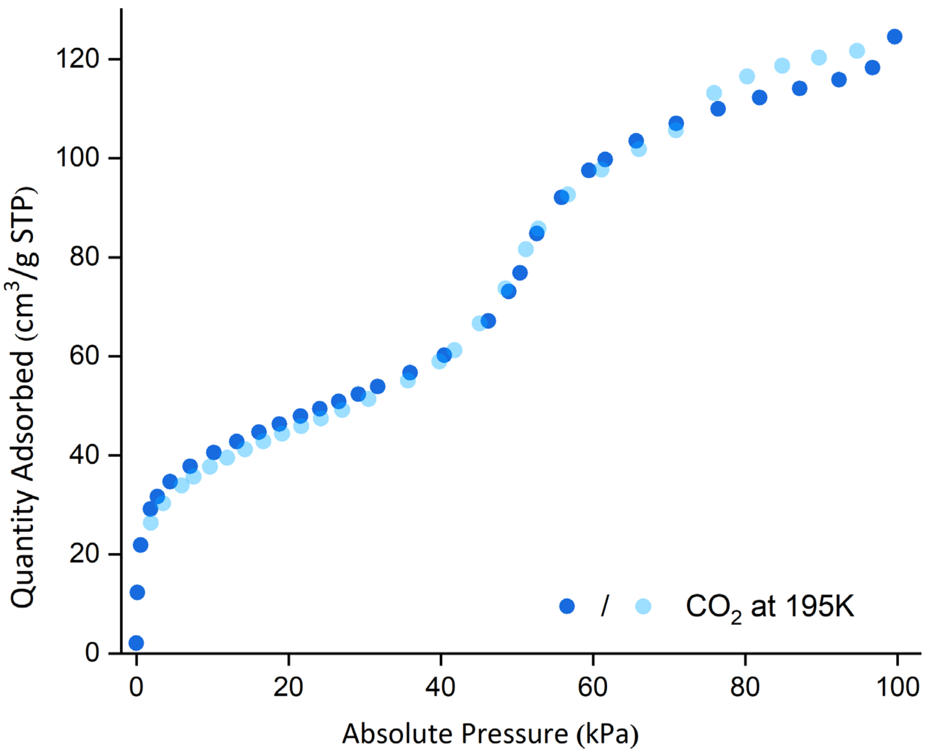

Supplementary Figure 18. CO_2 sorption isotherm measured at 195 K.

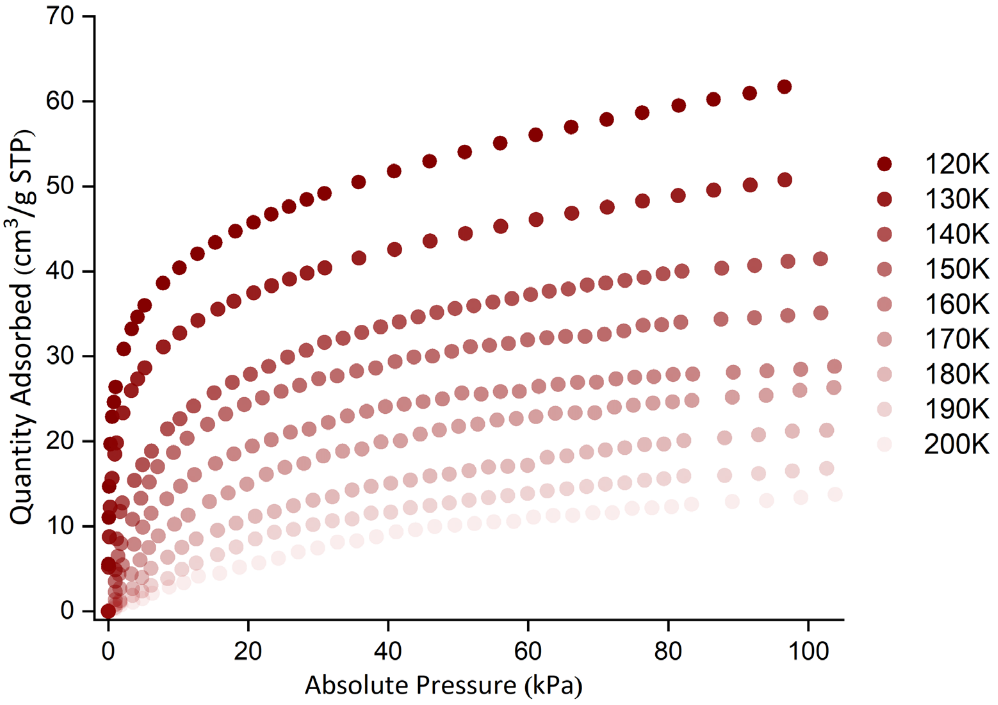

Supplementary Figure 19. Oxygen adsorption isotherms measured from 120 to 200 K in 10 °C interval of temperatures.

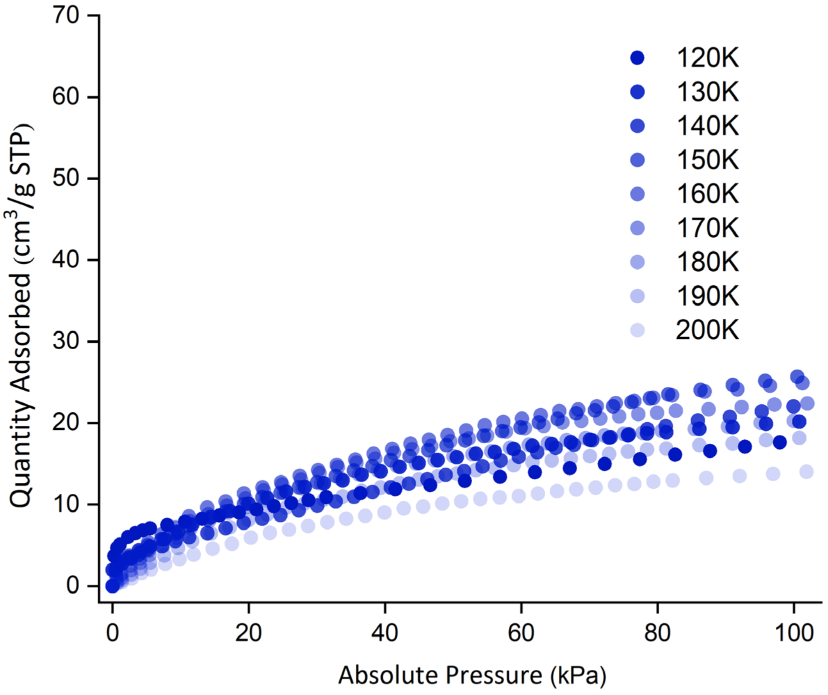

Supplementary Figure 20. Argon adsorption isotherms measured from 120 to 200 K in 10 °C interval of temperatures.

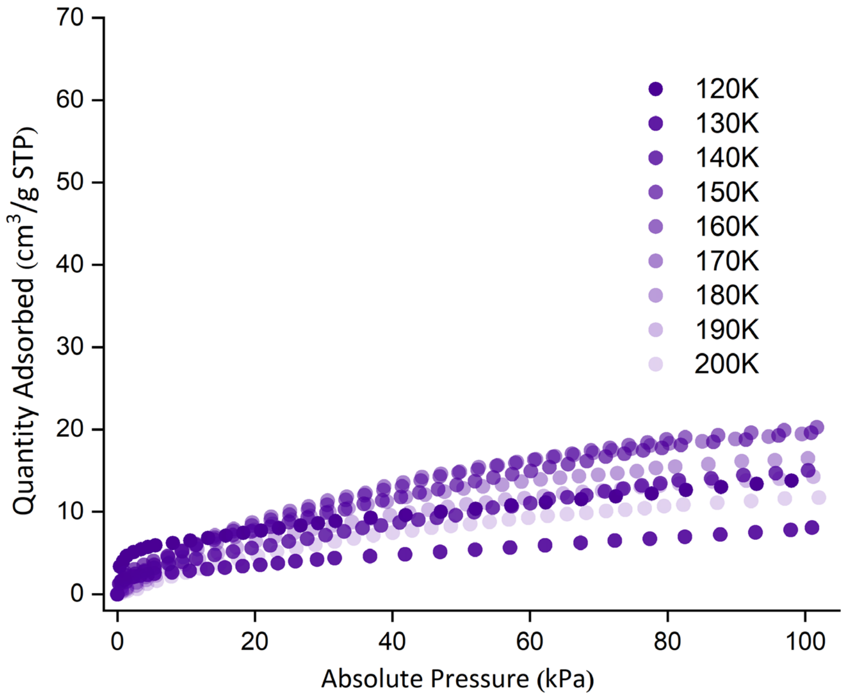

Supplementary Figure 21. Nitrogen adsorption isotherms measured from 120 to 200 K in 10 °C interval of temperatures.

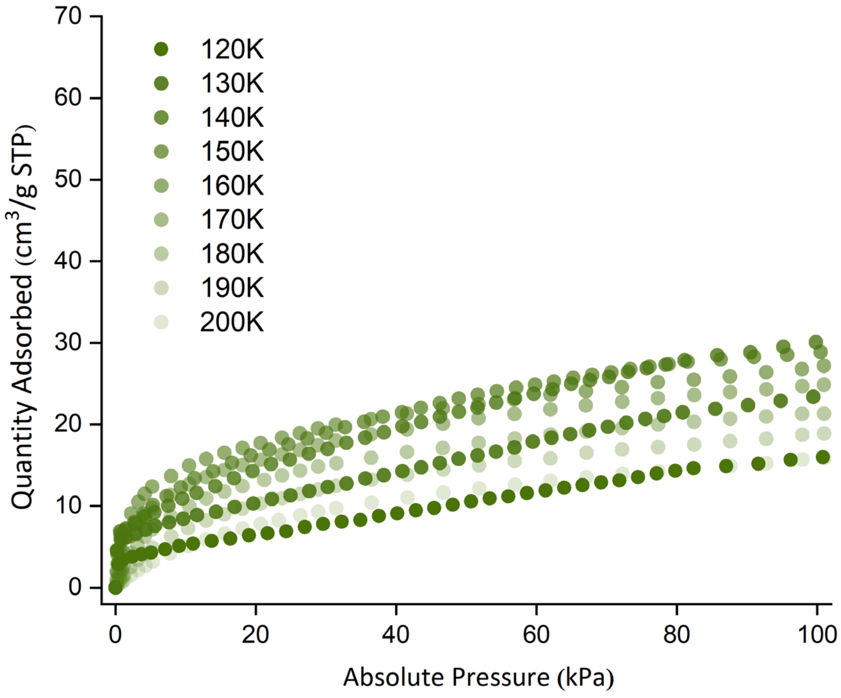

Supplementary Figure 22. Carbon monoxide adsorption isotherms measured from 120 to 200 K in 10 °C interval of temperatures.

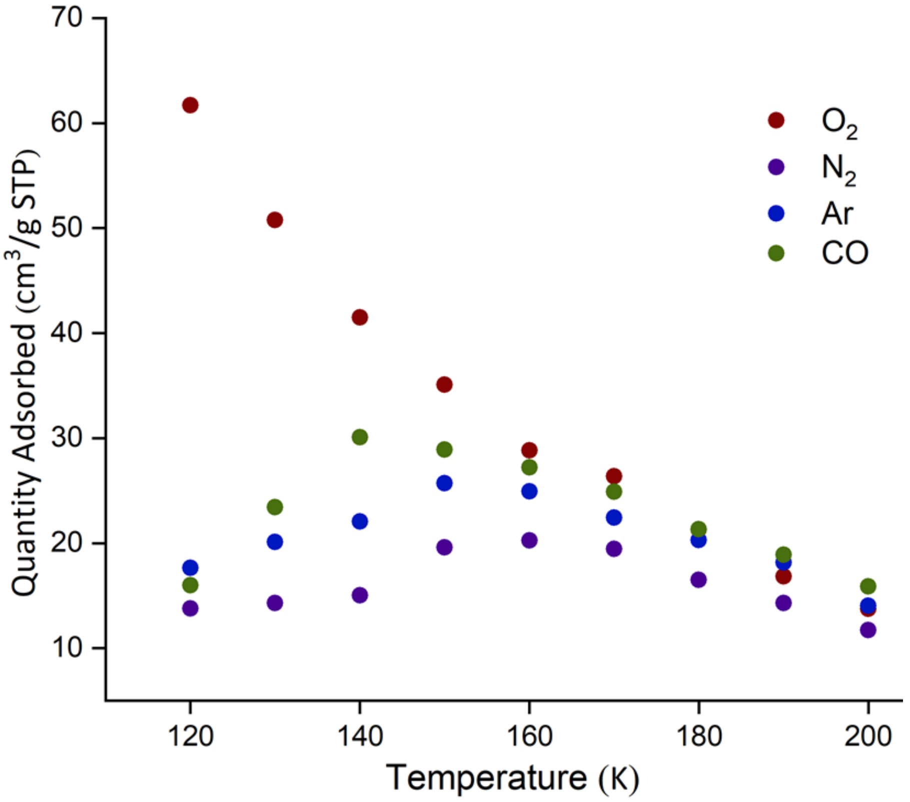

Supplementary Figure 23. Compared uptake at 1 bar for oxygen (red), nitrogen (purple), argon (blue) and carbon monoxide (green) from 120 to 200 K. Sorption measurements at above 120 K indicate that the O_2 uptake gradually decreases with the increase of the temperature, which is typical behavior of physisorbents. Whereas, uptake for Ar, N_2, and CO sorption increases gradually from 120 K to 140 K, and a further increase in temperature decrease the uptake capacity up to 200 K, indicating the diffusion-regulated type sorption mechanism.[1]

IAST Selectivities

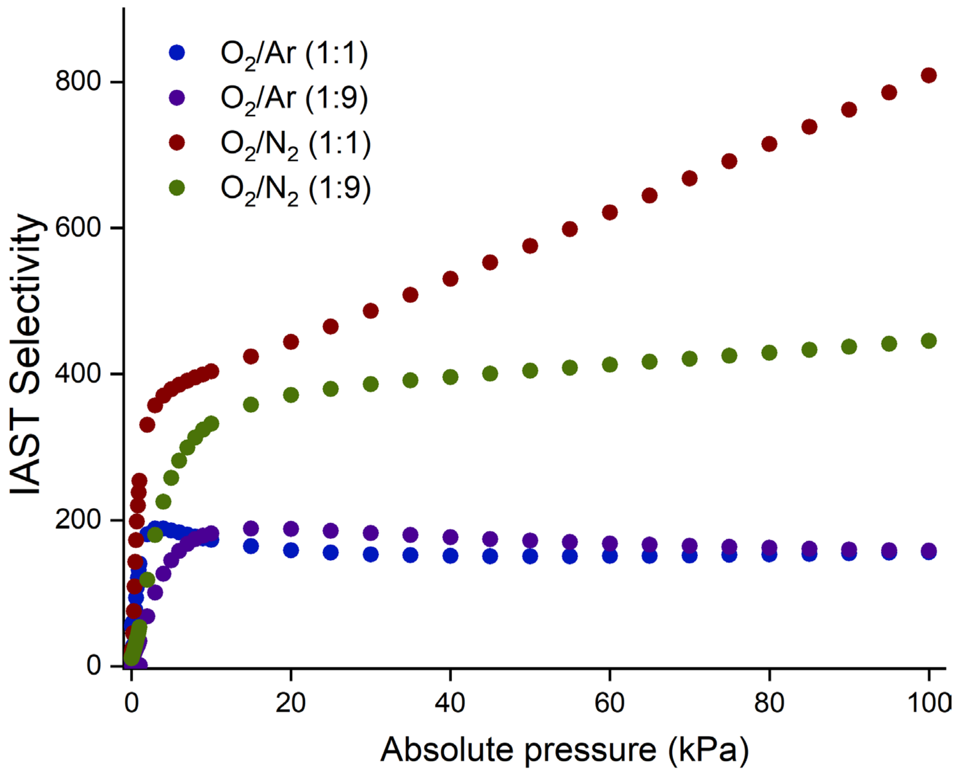

Supplementary Figure 24. IAST selectivities comparison for O_2/Ar, O_2/N_2 at 120 K and different concentrations.

Q_{st} plots for O_2, N_2 and Ar

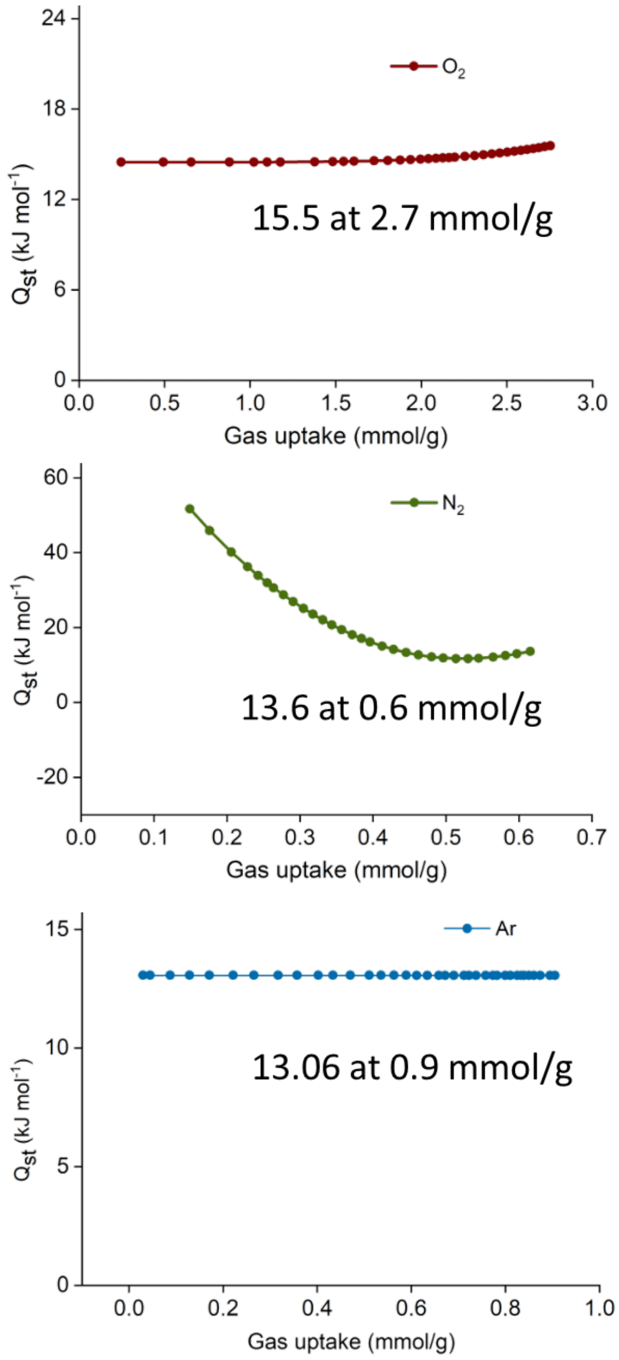

Supplementary Figure 25. Qst plots for O_2, N_2, and Ar as shown. The calculated Qst values are 15.5 at 2.7 mmol/g, 13.6 at 0.6 mmol/g, and 13.6 at 0.9 mmol/g for O_2, N_2, and Ar, respectively. In the case of O_2 and N_2, 120 and 130 K sorption isotherms were considered, while for Ar, 190 and 200 K sorption were fitted because 120 K is lower affinity than the 130 K sorption which is unusual behavior.

In-situ coincidence sorption and FT-IR measurements

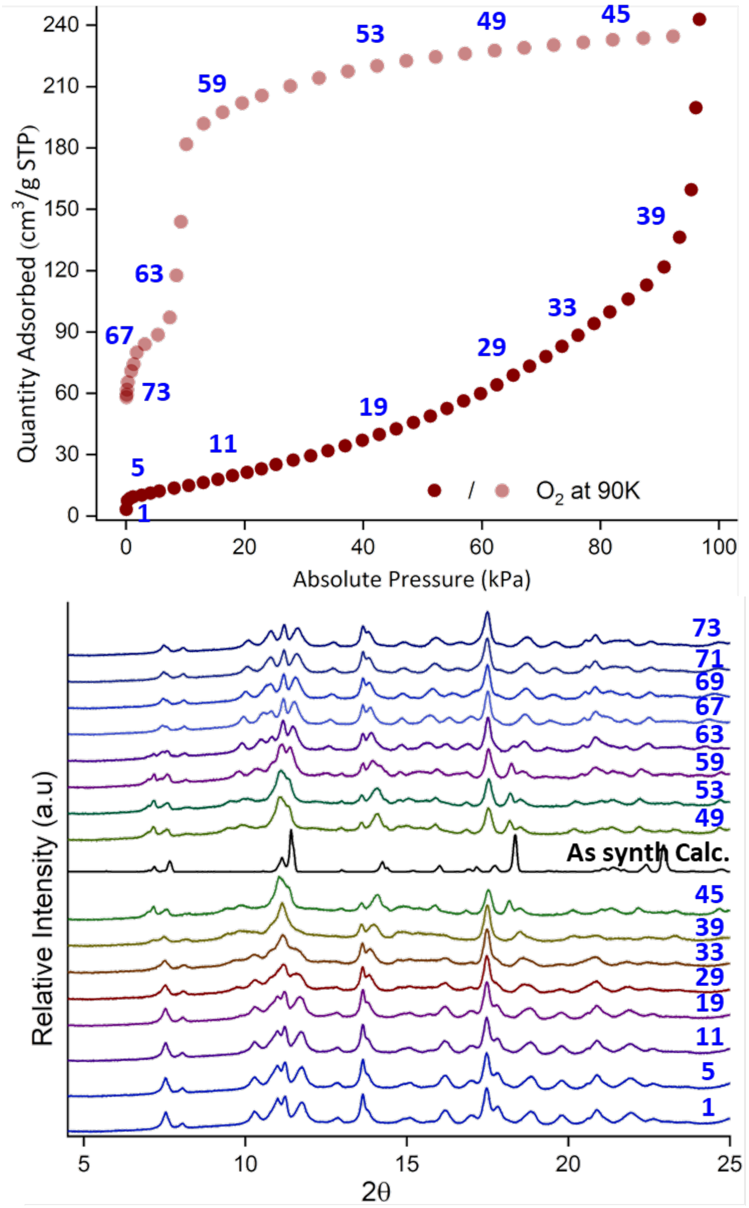

Supplementary Figure 26. In-situ PXRD measured upon sorption of O_2 at 90 K.

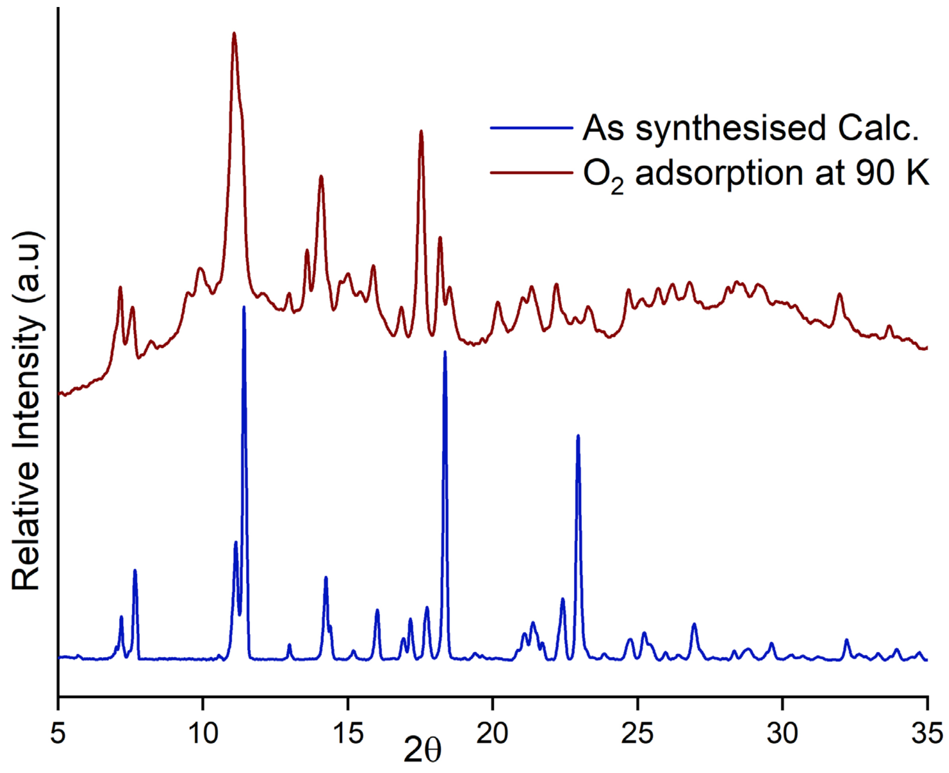

Supplementary Figure 27. The PXRD comparsions of as synthesized (calc.) vs. O_2 adsorbed phase (90 K). Which indicated that a phase obtained at fully O_2 loaded (100 Kpa) is similar to as synthesized calculated pattern except some small intensity peaks arises due to incomplete phase transformation from activated.

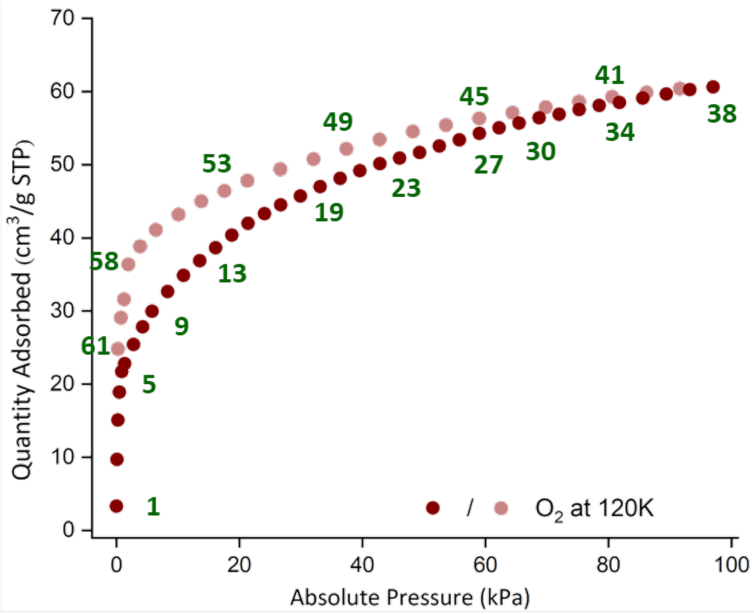

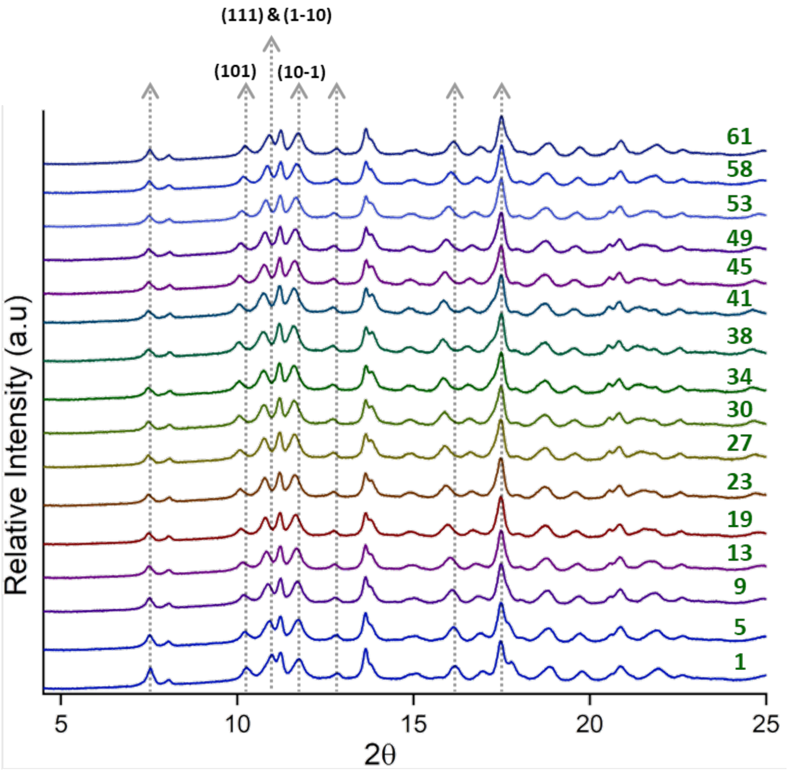

Supplementary Figure 28. In-situ PXRD measured upon sorption of O_2 at 120 K. The PXRD patterns underwent some shift in the peaks that corresponds to (101, 111, 1-10, and 10-1). The peak at 11 2θ is broad and spitted into two (please refer cif file).

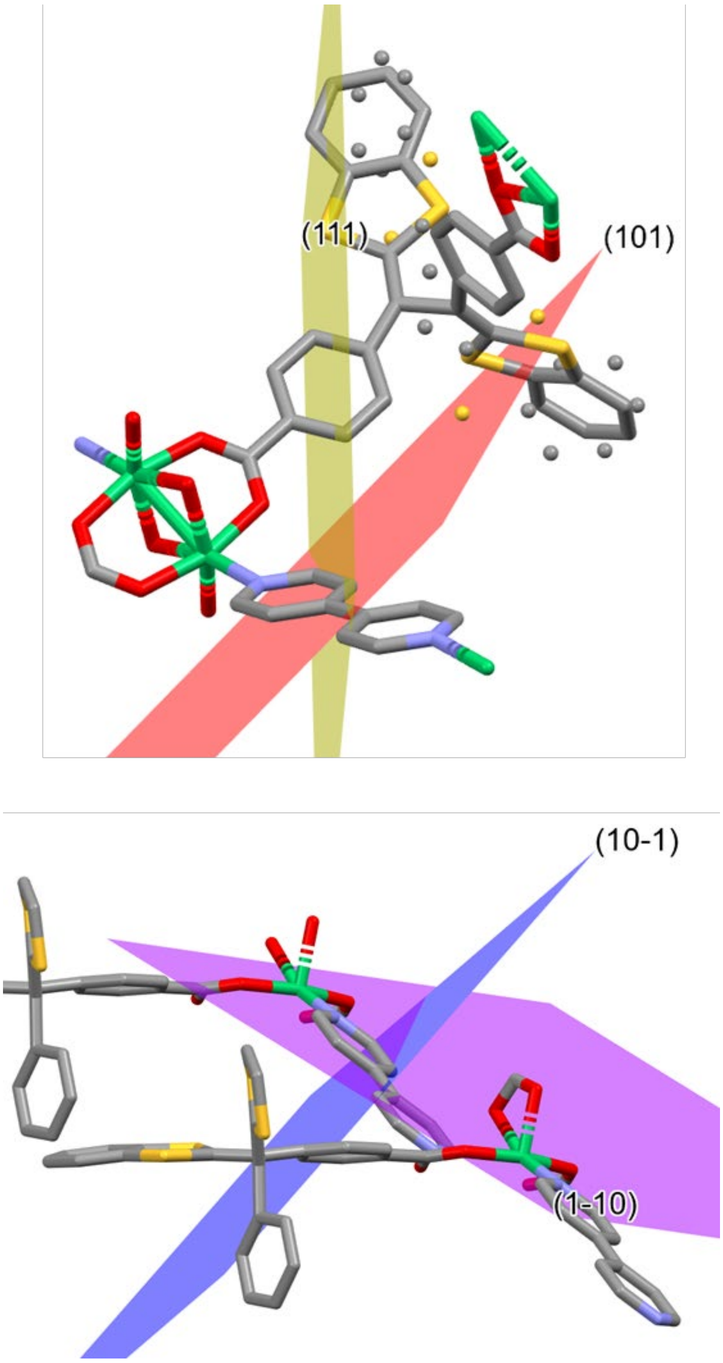

Supplementary Figure 29. The shifted peaks during O_2 sorption at 120 K that corresponds to (101), (111), (1-10), and (10-1) planes (Green, red, blue, yellow, gray spheres represent Ni, O, N, S and C, respectively).

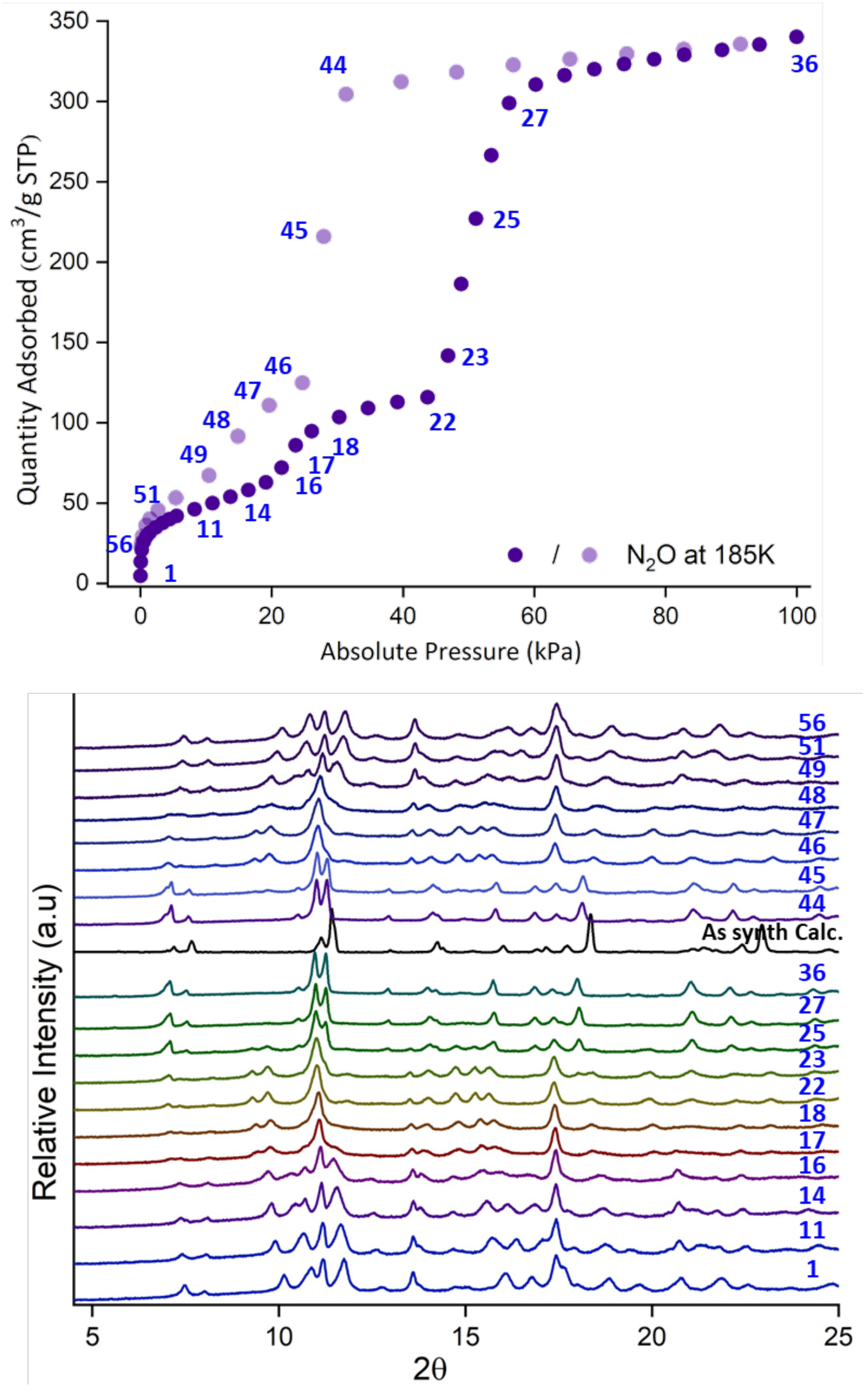

Supplementary Figure 30. In-situ PXRD measured upon sorption of N_2O at 185 K.

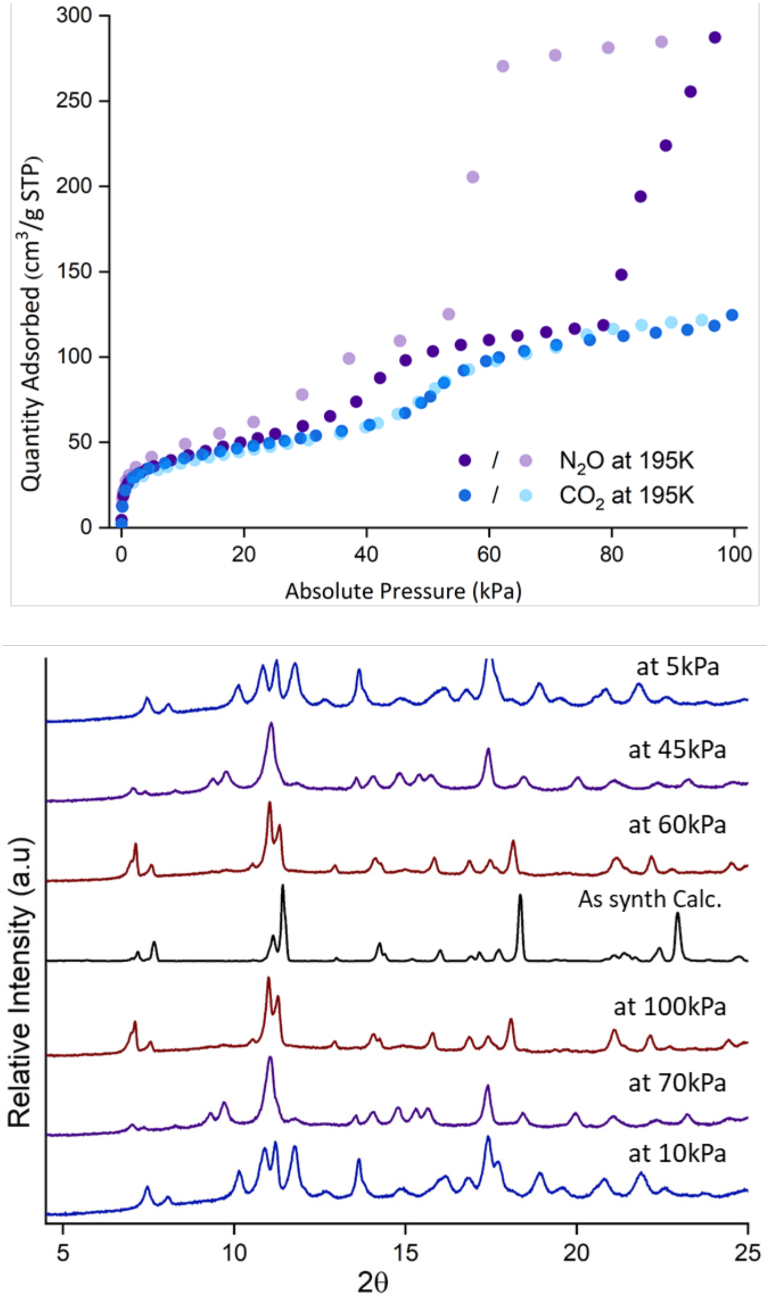

Supplementary Figure 31. In-situ PXRD measurements for N_2O sorption at 195 K and compared with CO_2 sorption profile at 195 K.

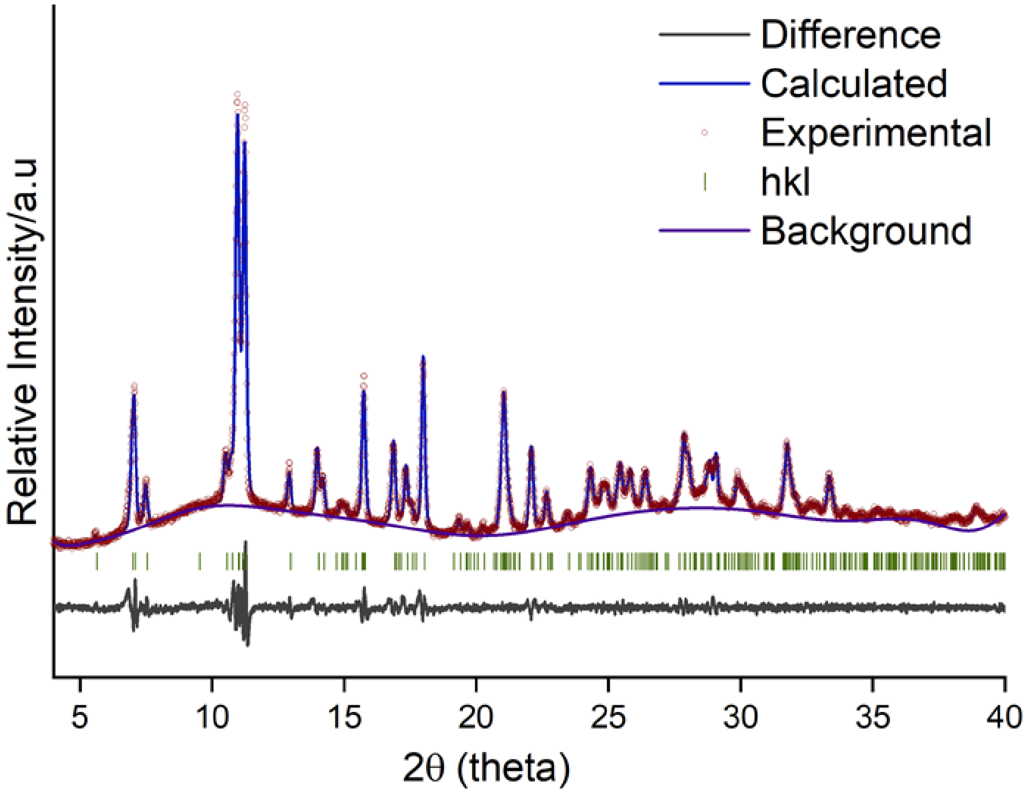

Supplementary Figure 32. The refinement results of PXRD for the N_2O fully loaded phase ($1\alpha \supset N_2O$) at 185 K obtained through pawley fitting. The experimental PXRD pattern (represented by a red circle) was compared to the calculated PXRD pattern (represented by blue), and the difference in the diffraction patterns was shown in black. The resulting cell parameters were slightly larger than those of the as-synthesized single crystal structure, indicating that the structure expanded upon fully loading N2O. Topas software was used for the calculation, and the space group and lattice parameters of the as-synthesized phase (C2/m, a = 33.09(11) Å, b = 11.887(4) Å, c = 13.470(5) Å, β = 111.49(8) º) were used as the starting point for the fitting. The refinement result gave the lattice parameters of a = 33.908(5) Å, b = 12.617(1) Å, and c = 13.643(7) Å, β = 112.604(7)º, and a cell volume of 5388.1(14) Å3, with Rp = 1.31% and Rwp = 1.04% (Rexp = 1.04).

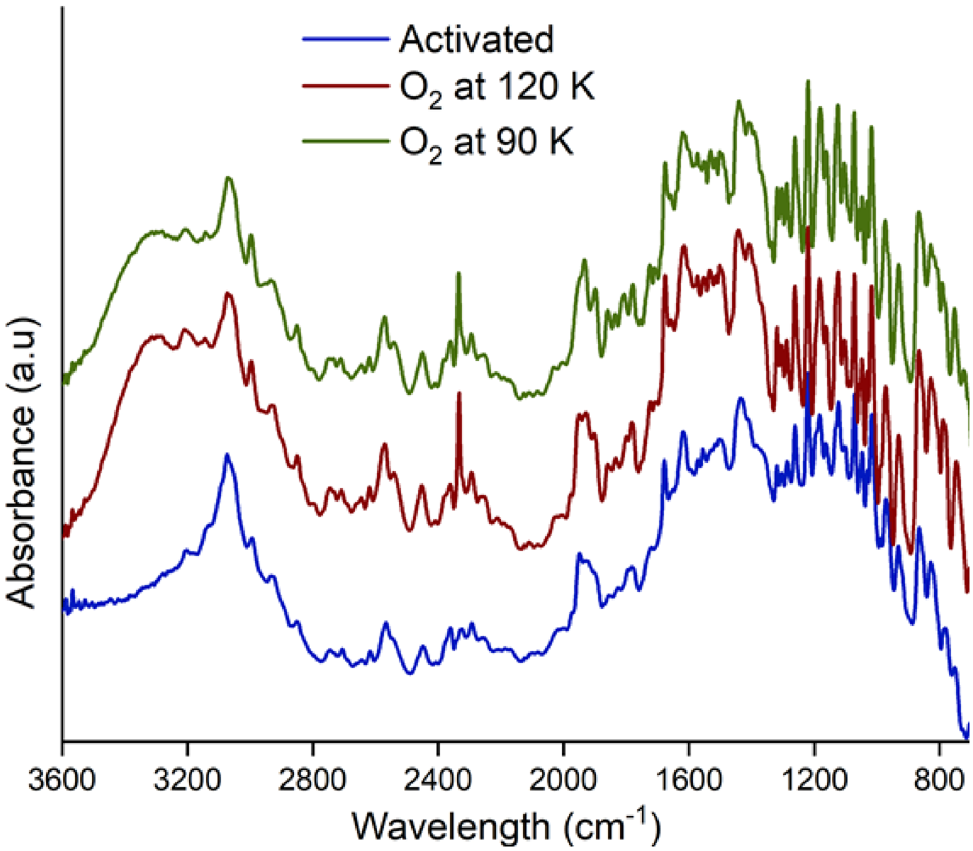

Supplementary Figure 33. In-situ FT-IR measurements upon O_2 sorption at 90 and 120 K.

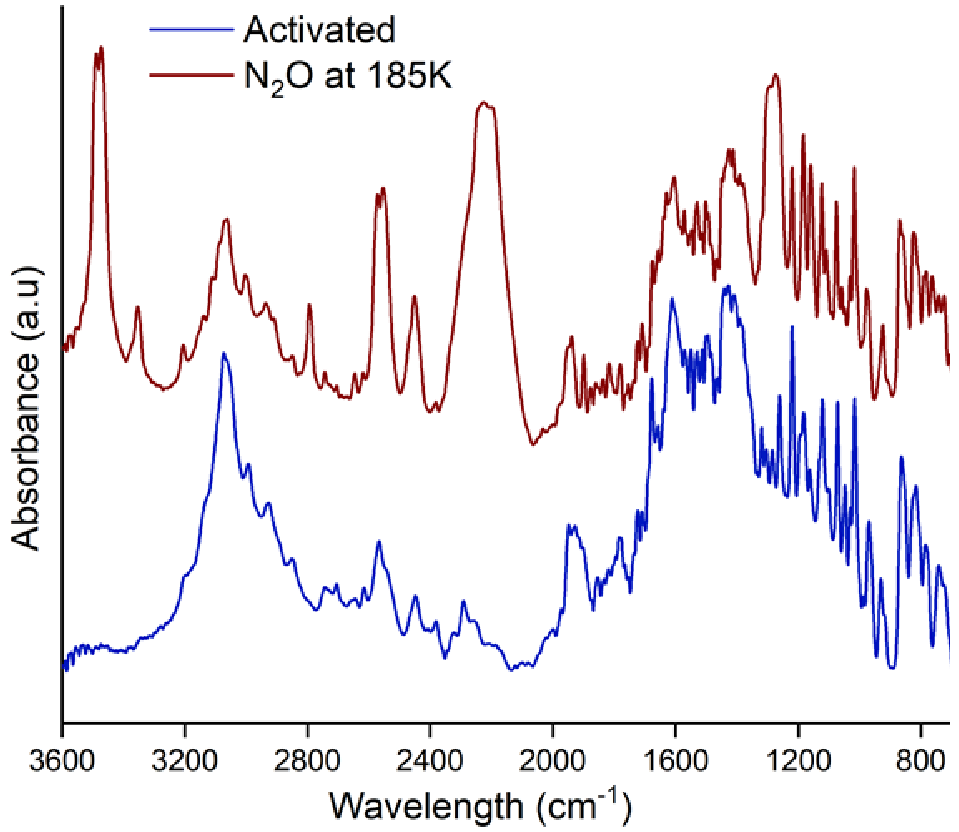

Supplementary Figure 34. In-situ FT-IR measurements upon N_2O sorption at 185 K.

Computational Details

The binding energies (BE) of gas molecules (G, G = Ar, N_2, and O_2) with $[Ni_2(4,4'\text{-bipyridine})(VTTF)_2]_n$ were calculated with eq. S1;

$$BE = E(PCP{\cdot}G)_{eq} - E(PCP)_{eq} - E(G)_{eq} \qquad (S1)$$

where E(PCP·G)eq is the total energy of $[Ni_2(4,4'\text{-bipyridine})(VTTF)_2]_n$ with one adsorbed gas molecule, E(PCP)eq and E(G)eq are the total energies of empty $[Ni_2(4,4'\text{-bipyridine})(VTTF)_2]_n$ and one free gas molecule, respectively, and the subscript "eq" represents the equilibrium structure. The BE was decomposed into the deformation energy (E_{def}) of PCP framework and the interaction energy (E_{int}) between gas molecule and PCP. E_{int} and E_{def} were calculated with eqs. S2 and S3;

$$E_{def} = E(PCP)_{dis} - E(PCP)_{eq} \qquad (S2)$$

$$E_{int} = E(PCP{\cdot}G)_{eq} - E(PCP)_{dis} - E(G)_{eq} \qquad (S3)$$

where $E(PCP)_{dis}$ is the total energy of $[Ni_2(4,4'\text{-bipyridine})(VTTF)_2]_n$ in the distorted (dis) geometry induced by gas adsorption, taked from the optimed geometry of $[Ni_2(4,4'\text{-bipyridine})(VTTF)_2]_n$ with one adsorbed gas molecule. The deformation energy of gas molecule is very small and thus was neglected.

The zero-temperature formation energies of the N_2O loaded 1α and 1β phases were calculated with DFT calculations of the relevant phase with 0, 0.5, 5.5, and 11 adsorbates (N_2O molecules) per framework Ni atom in the unit cell of the calculation (11 adsorbates per Ni atom only fit in the 1α phase). The energies were then normalized to the number of framework Ni atoms in the cell for comparison between the differing unit cell sizes for the two phases.

Supplementary Table 4. Localized molecular orbital energy decomposition analysis[a] on the interaction energy (kcal mol^{-1}) of gas molecule with several fragments around the adsorbed gas molecule in [Ni_2(4,4'-bipyridine)(VTTF)$_2$]$_n$ at site I.

Energy term	Fragment 1			Fragment 2			Fragment 3		
	Ar	N_2	O_2	Ar	N_2	O_2	Ar	N_2	O_2
$E_{PBE–D3}$	–1.4	–1.7	–2.2	–1.4	–1.8	–1.8	–1.1	–1.1	–1.3
E_{DIS}	–3.9	–3.7	–4.0	–3.4	–3.6	–3.4	–3.9	–4.2	–3.7
E_{ES}	–1.7	–1.3	–1.8	–1.4	–1.3	–1.6	–1.6	–1.4	–1.6
E_{EXR}	4.9	4.1	5.4	4.2	4.6	4.8	5.1	5.4	5.0
$E_{CT+Pol+Mix}$	–0.7	–0.8	–1.7	–0.8	–1.0	–1.7	–0.7	–1.0	–1.2

[a] Localized molecular orbital energy decomposition analysis[2] was carried out with aug-cc-PVDZ basis set using the GAMESS package.[3] Cluster models shown in Figure S36 were used in these calculations.

[b] The total interaction energy (E_{int}) was calculated at the PBE–D3 level (E_{PBE-D3}), which consists of the dispersion energy (E_{DIS}), electrostatic interaction (E_{ES}), exchange repulsion (E_{EXR}), and charge transfer, polarization, and mixing terms ($E_{CT+Pol+Mix}$).

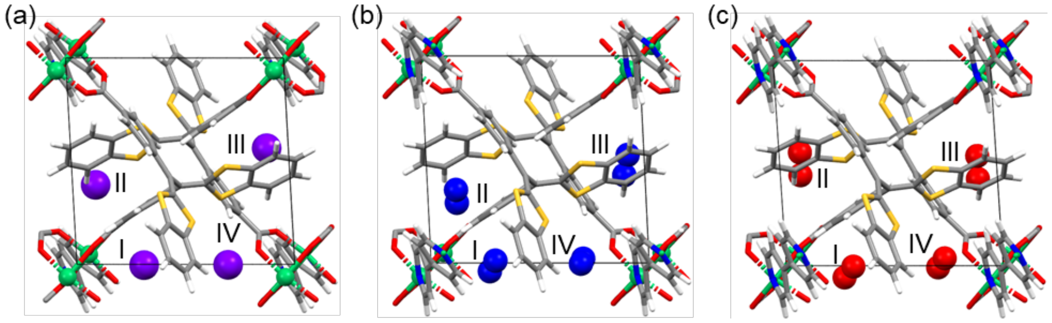

Supplementary Figure 35. Computation–revealed plausible gas adsorption positions for (a) Ar, (b) N_2, and (c) O_2 in the β phase (Green, red, blue, yellow, gray spheres represent Ni, O, N, S and C, respectively).

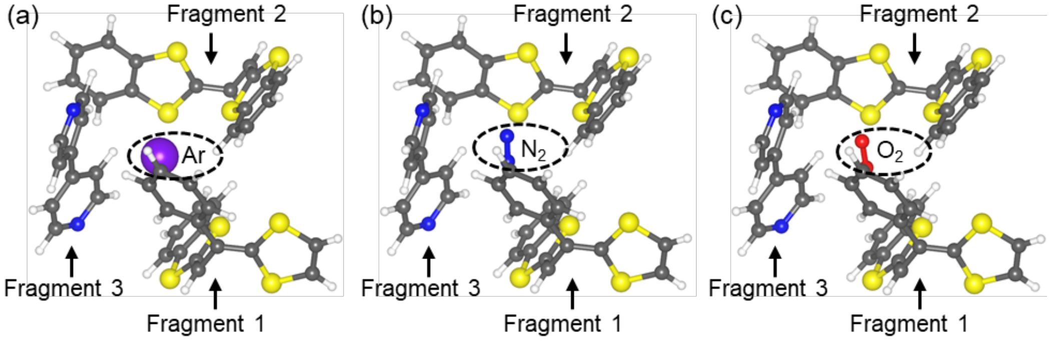

Supplementary Figure 36. Cluster models used in energy decomposition analysis on the interaction energy between gas molecule and PCP framework of β phase.

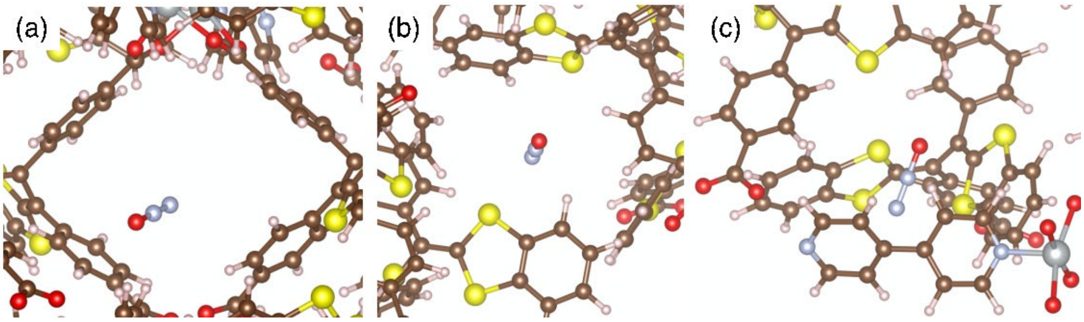

Supplementary Figure 37. Binding sites analyzed for N_2O and O_2 in the PCP 1α phase: (a) site 1, (b) site 2, and (c) site 3. (N_2O shown, O_2 sites are similar).

Supplementary References

[1] C. Gu, *et al. Science* **2019**, 363, 387–391.

[2] P. Su, H. Li, *J. Chem. Phys.* **2009**, *131*, 014102.

[3] M. W. Schmidt, K. K. Baldridge, J. A. Boatz, S. T. Elbert, M. S. Gordon, J. H. Jensen, S. Koseki, N. Matsunaga, K. A. Nguyen, S. J. Su, T. L. Windus, M. Dupuis, J. A. Montgomery, *J. Comput. Chem.* **1993**, *14*, 1347–1363.
