# Supplementary material for: Selective sorption of oxygen and nitrous oxide by an electron donor-incorporated flexible coordination network
Source: Commun Chem. 2023 Apr 4;6:62. doi: 10.1038/s42004-023-00853-1 (PMC10073098; doi:10.1038/s42004-023-00853-1)
Supplement: Supplementary file 2 — Description of Additional Supplementary Files [file 42004_2023_853_MOESM2_ESM.pdf]

Description of Additional Supplementary Files

File name: Supplementary Data 1

Description: Incar file for VASP calculations

File name: Supplementary Data 2

Description: Coordinates for optimized adsorption structures of different gas molecules in 1β phase

File name: Supplementary Data 3

Description: CIF file for 1α phase

File name: Supplementary Data 4

Description: CIF file for 1β phase

File name: Supplementary Data 5

Description: CIF file for 1α⊃3C6H6 phase
